# Effects of Short-Term Dietary Sodium Selenite Supplementation on Antioxidant Status, Heavy Metal Deposition, Flesh Quality and Flavor-Related Compounds of Common Carp (*Cyprinus carpio*), and a Potential Mechanism

**DOI:** 10.3390/antiox15101233

**Published:** 2026-09-24

**Authors:** Wanwen Chen, Scovia Kikoberwa, Jian Wu, Pao Xu, Hao Cheng, Haibo Wen

**Affiliations:** 1 Wuxi Fisheries College, Nanjing Agricultural University, Wuxi 214081, China; chenwanwen@ffrc.cn (W.C.);; 2 Key Laboratory of Integrated Rice-Fish Farming Ecology, Ministry of Agriculture and Rural Affairs, Freshwater Fisheries Research Center, Chinese Academy of Fishery Sciences, Wuxi 214081, China; 3 Sino-US Cooperative International Laboratory for Germplasm Conservation and Utilization of Freshwater Mollusks, Freshwater Fisheries Research Center, Chinese Academy of Fishery Sciences, Wuxi 214081, China; 4 State Key Laboratory of Food Science and Resources, School of Food Science and Technology, Jiangnan University, Wuxi 214122, China

**Keywords:** sodium selenite, common carp, antioxidant capacity, flesh quality, volatile compounds

## Abstract

Sodium selenite is a cost-effective selenium source widely used in aquafeed, yet its effects on the flesh quality of adult common carp (*Cyprinus carpio*) remain poorly understood. Using a short-term feeding trial simulating pre-harvest selenium biofortification, this study investigated dietary sodium selenite effects on growth, antioxidant status, selenium deposition, heavy metal accumulation, nutritional composition, and flavor-related compounds in adult common carp. Dietary selenium supplementation improved weight gain and specific growth rates, with optimal doses ranging from 1.797 to 1.804 mg Se kg^−1^. Elevated hepatic and muscular glutathione peroxidase (GSH-Px) and catalase (CAT) activities, coupled with decreased malondialdehyde (MDA) concentrations, were closely associated with increased selenium deposition in muscle. Selenium deposition was also correlated with decreased accumulation of heavy metals (Hg and Cd), which may be attributable to the formation of inert complexes. The improved antioxidant status was further linked with better muscle texture and water-holding capacity. Moreover, dietary sodium selenite increased muscle crude protein content and the abundance of umami-active free amino acids. The proportional content of total eicosapentaenoic acid (EPA) and docosahexaenoic acid (DHA) increased from 3.55% to 5.14%, accompanied by alterations in flavor-related volatile compounds. Untargeted metabolomic analysis further revealed that dietary selenium supplementation substantially modulates lipid metabolism, as well as PPAR and FoxO signaling pathways. Collectively, these findings offer experimental insights supporting the development of selenium-enriched common carp production under short-term pre-harvest fortification strategies.

## 1. Introduction

Fish represent ideal biological carriers for mineral and nutrient biofortification, owing to their high-quality protein, health-promoting unsaturated fatty acids, and superior mineral bioavailability [1]. Selenium (Se) is an indispensable trace element for both humans and aquatic animals, and its biological functions are mainly mediated by selenoproteins. In the fish antioxidant system, key selenoproteins, including glutathione peroxidases (GPXs), thioredoxin reductases (TrxRs), selenoprotein P (SELENOP), and selenoprotein S (SELENOS), play pivotal roles in regulating oxidative stress induced by reactive oxygen species (ROS), such as hydrogen peroxide (H_2_O_2_) and other free radicals. This selenoprotein-dependent antioxidant defense is critical for maintaining redox homeostasis and regulating lipid peroxidation, thereby underscoring its essential role in preserving fish health and flesh quality [2,3]. Despite its essential physiological roles, Se deficiency remains a severe public health issue in China, affecting more than 70 million people across approximately 72% of the national land area [4]. Although Se naturally exists in soil, its low bioavailability severely restricts Se enrichment in aquaculture systems. Therefore, dietary Se biofortification has become a practical and sustainable strategy to improve Se nutritional status and promote public health [5].

In aquaculture practice, Se is essential for maintaining normal growth, physiological development, and systemic health of fish [6]. Notably, Se can effectively antagonize the bioaccumulation of toxic heavy metals, which is critically important for ensuring seafood safety. Both Se deficiency and oversupply exert detrimental effects, leading to growth retardation, muscle injury, oxidative stress, metabolic disorders, or even acute toxicity [7,8]. The optimal dietary Se level varies substantially among fish species, developmental stages, and chemical forms of Se [9]. Previous studies have established that the recommended dietary Se concentrations for most farmed fish range from 0.1 to 2.00 mg/kg. For instance, Atlantic salmon (*Salmo salar*) require 1–3 mg Se kg^−1^, whereas smaller fish species demand considerably lower levels [10,11]. However, the vast majority of existing studies have focused on growth performance, feed utilization, Se accumulation, antioxidant capacity, hematological parameters, and immune responses in juvenile fish [12,13,14], with very limited attention paid to the effects of Se on fish flesh quality traits in adult fish. Specifically, the understanding of how sodium selenite influences flesh quality in adult fish through redox regulation, lipid metabolism pathways, and its interactions with heavy metal accumulation remains a critical knowledge gap that needs to be addressed.

Common carp (*Cyprinus carpio* L.) is a dominant freshwater aquaculture species in Asia, accounting for 10.3% of total freshwater fish production in China in 2024, highlighting its high economic and nutritional importance [15]. Given that dietary selenium supplementation significantly elevates selenium deposition in its muscle [16], common carp represents a promising candidate for Se biofortification. Sodium selenite is the most widely used inorganic Se source in commercial aquafeeds due to its low cost, high stability, and ease of large-scale application. Nevertheless, comprehensive information regarding the effects of sodium selenite supplementation on flesh quality of adult common carp, including nutritional composition, textural properties, taste-active components, volatile flavor, and underlying metabolic mechanisms, remains largely unknown. To address this gap, the present study integrates graded dietary sodium selenite supplementation with untargeted metabolomics to establish a comprehensive link among redox status, lipid metabolism, amino acid profiles, fatty acid composition, and volatile compounds in adult common carp.

Accordingly, the present study was conducted to systematically investigate the effects of graded dietary sodium selenite (0, 0.5, 1.0, and 2.0 mg Se kg^−1^) on growth performance, antioxidant status, tissue Se deposition, heavy metal accumulation, flesh physical traits, fatty acid profiles, free amino acid composition, and volatile flavor compounds in adult common carp. Untargeted metabolomics was further employed to reveal the key metabolic pathways and molecular mechanisms related to lipid metabolism and redox regulation. This study aims to provide a scientific basis for short-term Se biofortification to enhance the quality, safety, and nutritional value of farmed carp, and support the development of high-quality Se-enriched freshwater fish products.

## 2. Materials and Methods

### 2.1. Feeding Condition and Sample Collection

#### 2.1.1. Experimental Diets

The ingredient composition of the basal diet is shown in Table S1. Sodium selenite (Sigma-Aldrich, St. Louis, MO, USA) was incorporated into the basal diet via step-by-step dilution to obtain nominal dietary selenium concentrations of 0.5, 1.0, and 2.0 mg Se kg^−1^ diet. The unsupplemented basal diet served as the control group. All experimental diets were fully mixed, extruded into pellets, air-dried at ambient temperature, and stored at −20 °C prior to use. Actual selenium concentrations in the finished diets were determined using inductively coupled plasma-mass spectrometry (ICP-MS, Agilent 7500CE, Agilent Technologies, Inc., Santa Clara, CA, USA), yielding measured values of 0.61, 1.20, 1.98, and 2.55 mg Se kg^−1^ diet for the control, 0.5, 1.0, and 2.0 mg Se kg^−1^ groups, respectively.

#### 2.1.2. Experimental Design

Healthy adult common carp (*Cyprinus carpio*) were obtained from the Freshwater Fisheries Research Center, Chinese Academy of Fishery Sciences (Wuxi, China). Prior to the experiment, fish were acclimated in tanks and fed to satiation twice daily with a commercial diet for 2 weeks. A total of 240 fish with an average initial weight of 250 ± 15 g were randomly allocated into 12 net cages, with three replicate cages per treatment group. Fish were hand-fed twice daily (8:00 and 17:00) for 8 weeks. Water temperature was maintained at 24.0 ± 2.0 °C. Water quality parameters, including pH, ammonia nitrogen, nitrite, and dissolved oxygen, were monitored twice weekly and maintained within suitable ranges: pH 7.0–8.0, ammonia nitrogen < 0.5 mg/L, dissolved oxygen > 7.5 mg/L.

#### 2.1.3. Sample Collection

Following a 24 h fasting period, four fish per cage were randomly selected and euthanized following eugenol anesthesia. Dorsal muscle and liver tissues were rapidly excised, snap-frozen in liquid nitrogen, and preserved at −80 °C for subsequent biochemical and omics detection. Fresh dorsal muscle was subdivided into two aliquots. The first aliquot was kept at 4 °C and analyzed within 24 h to determine muscle texture and water-holding capacity. For the second aliquot, tissues from four fish originating from the same cage were pooled to generate one composite sample per cage, resulting in three composite samples for each treatment group. These pooled composite samples, preserved at −20 °C, were used for measurements of selenium content, selenium speciation, nutritional composition, and volatile flavor-related compounds.

### 2.2. Biochemical Properties Analysis

#### 2.2.1. Growth Performance Analysis

Growth performance was assessed by recording initial weight (*W*_I_), final weight (*W*_F_), body length (*L*_B_), liver weight (*W*_L_), visceral mass (*W*_V_), and the total experiment duration (T). The weight gain rate (WGR), specific growth rate (SGR), hepatosomatic index (HSI), viscerosomatic index (VSI), and condition factor (CF) were calculated as follows:

(1) $$\mathrm{WGR}\;(\%)={(W}_{F}-W_{I})/W_{I}\times 100$$

(2) $$\mathrm{SGR}\;(\%/\mathrm{day})={\mathrm{In}\;(W}_{F}/W_{I})/T\times 100$$

(3) $$\mathrm{HSI}\;(\%)=W_{L}/W_{F}\times 100$$

(4) $$\mathrm{VSI}\;(\%)=W_{V}/W_{F}\times 100$$

(5) $$\mathrm{CF}\;(g/{\mathrm{cm}}^{3})=W_{F}/{L_{B}}^{3}\times 100$$

#### 2.2.2. Flesh Composition Analysis

Proximate composition of muscle, including moisture, crude protein, crude lipid, and ash, was determined according to the standard methods of the Association of Official Analytical Chemists (AOAC) [17]. Specifically, moisture was measured by drying at 105 °C to constant weight. Crude protein was analyzed using the Kjeldahl method. Ash content was determined via incineration at 550 °C for 12 h. Crude lipid was extracted using the Soxhlet method.

#### 2.2.3. Antioxidant Enzyme Activity Analysis

Muscle or liver samples (0.1 g) were homogenized in 0.9 mL of physiological saline using a high-throughput tissue grinder (Scientz-48L, Xinzhi Biotechnology Co., Ltd., Ningbo, China). Homogenates were centrifuged at 3000 rpm at 4 °C for 10 min, and supernatants were collected for subsequent analysis of glutathione peroxidase (GSH-Px), superoxide dismutase (SOD), catalase (CAT), and malondialdehyde (MDA) levels. The activities of GSH-Px, SOD, and CAT were determined by colorimetric, hydroxylamine, and ammonium molybdate chromogenic methods, respectively, while MDA concentration was measured using the thiobarbituric acid (TBA) method.

### 2.3. Selenium Concentration and Heavy Metal Antagonism Analysis

#### 2.3.1. Selenium Concentration Analysis

Selenium concentrations in muscle and liver were determined using inductively coupled plasma mass spectrometry (ICP-MS, Agilent 7500CE, Agilent Technologies, Inc., Santa Clara, CA, USA) according to the previous method with minor modifications [18]. Briefly, tissue samples were digested with nitric acid using an electric heating digestion apparatus (Model LC-DB-3DA, LICHEN, Lichen Scientific Instruments Co., Ltd., Shaoxing, Zhejiang, China). A programmed heating procedure was set as follows: the temperature was ramped from room temperature to 100 °C over 10 min and held for 10 min; subsequently increased to 120 °C over 10 min with a 10 min hold; and finally raised to 200 °C over 10 min, followed by continuous digestion at 200 °C for 60 min. The final digest was concentrated, cooled to room temperature, diluted with ultrapure water, and filtered through a 0.22 μm membrane. Subsequently, the digests were analyzed by ICP-MS with high-purity argon as the plasma gas. The operating conditions were as follows: radio frequency power, 1550 W; radio frequency matching, 1.80 V; auxiliary gas flow rate, 1.5 L/min; carrier gas flow rate, 0.71 L/min. Method validation was performed for Se determination.

#### 2.3.2. Heavy Metal Antagonism Analysis

Muscle concentrations of mercury (Hg) and cadmium (Cd) were determined using ICP-MS under the same instrumental conditions as selenium analysis to evaluate the antagonistic effect of selenium. Matrix-certified reference material (CRM, GBW10051) was routinely analyzed as a quality control sample to verify the accuracy and precision of Se, Cd, and Hg measurements. The certified values were Se: 1.54 ± 0.29, Cd: 1.00 ± 0.07, and Hg: 0.045 ± 0.008 mg/kg. The obtained values of CRM were within the certified range, confirming acceptable method performance. Precision was evaluated as the relative standard deviation (RSD, *n* = 3) based on replicate analyses of the CRM, with RSD of 1.94% for Se, 1.24% for Cd, and 2.08% for Hg. The limit of detection (LOD) and quantification (LOQ) of total Se, Cd and Hg were calculated with signal-to-noise ratios (S/N) of 3 and 10, respectively: Se 0.04/0.12, Cd 0.03/0.10, Hg 0.09/0.30 µg/kg.

### 2.4. Flesh Quality Analysis

#### 2.4.1. Texture Profile Analysis

For texture profile analysis (TPA), dorsal muscle was cut into cubes (2 cm × 2 cm × 1 cm) and analyzed with a TA-XTPlus texture analyzer (Stable Micro Systems, Surrey, UK) equipped with a P/5 cylindrical probe. The test conditions were as follows: pre-test speed, 2 mm/s; test speed, 1 mm/s; post-test speed, 2 mm/s; trigger force, 5 g; interval, 10 s; and deformation, 60%. The parameters recorded were hardness, springiness, cohesiveness, gumminess, chewiness, and resilience.

#### 2.4.2. Centrifugal Loss Analysis

Water-holding capacity was evaluated by measuring centrifugal loss according to the method with slight modifications [19]. Briefly, an accurately weighed muscle sample (*W*_O_) was wrapped in filter paper, placed in a centrifugal tube, and centrifuged at 4000 rpm for 30 min at 4 °C. After centrifugation, the sample was removed, surface moisture was absorbed with filter paper, and the sample was reweighed (*W*_F_). The centrifugal loss was calculated using the following equation:

(6) $$\mathrm{Centrifugal}\;\mathrm{loss}\;(\%)={(W}_{O}-W_{F})/W_{F}\times 100$$

### 2.5. Nutritional, Taste and Volatile Flavor Analysis

#### 2.5.1. Hydrolyzed Amino Acid Analysis

The hydrolyzed amino acids were determined using an S433D automatic amino acid analyzer (Sykam, Eresing, Germany). Briefly, 0.1 g of muscle sample was accurately weighed and mixed with 6 M HCl in a hydrolysis tube. After purging with nitrogen for 3 min to evacuate oxygen, the tube was sealed and subjected to hydrolysis at 120 °C for 24 h. Following hydrolysis, the sample was neutralized with 10 M NaOH and diluted to a final volume of 25 mL with distilled water. The solution was filtered through double-layer filter paper and centrifuged at 10,000 rpm for 10 min. Subsequently, 400 μL of the supernatant was passed through a 0.22 μm aqueous-compatible membrane filter prior to analysis. Amino acid composition was expressed as a percentage of total protein.

#### 2.5.2. Fatty Acid Analysis

The fatty acid composition was determined using a GC-2030AF gas chromatograph (Shimadzu, Kyoto, Japan), following a previously described procedure with minor modifications [20]. Briefly, total crude lipid was extracted overnight using chloroform-methanol (2:1, *v*/*v*). For methyl ester derivatization, the extracted lipid was reacted with 0.5 M sodium hydroxide in methanol at 60 °C for 30 min. After cooling, boron trifluoride in methanol was added, and the mixture was incubated at 60 °C for a further 30 min. Subsequently, n-hexane was added to extract the fatty acid methyl esters, and the upper organic layer was collected. This layer was subsequently filtered through a 0.22 μm membrane e prior to GC analysis. Individual fatty acid peaks were identified by matching retention times against certified mixed fatty acid standards. Fatty acid relative proportions were calculated via peak-area normalization, in which the peak area of each individual fatty acid was divided by the total peak area of all detected fatty acids, and results were expressed as a percentage of total detected fatty acids (% of total fatty acid).

#### 2.5.3. Free Amino Acid Analysis

The free amino acids were quantitatively determined using an S433D automatic amino acid analyzer (Sykam, Eresing, Germany). Briefly, 2 g of muscle sample was homogenized in 5% trichloroacetic acid (25 mL) and ultrasonicated for 30 min, followed by 3 h of incubation to precipitate proteins and release amino acids. Subsequently, the mixture was filtered and centrifuged at 10,000× *g* for 10 min. The supernatant was finally filtered through a 0.22 μm membrane prior to analysis.

#### 2.5.4. Volatile Compounds Analysis

Volatile compounds in common carp muscle were extracted by headspace solid-phase microextraction (HS-SPME), following a previously reported method with slight modifications [21]. Briefly, 1.5 g of muscle sample was homogenized with 6 mL of saturated sodium chloride solution and transferred into a headspace vial. A total of 15 μL of 100 μg/mL 2, 4, 6-trimethylpyridine solution was added as an internal standard. The SPME fiber (65 μm, PDMS/DVB; Supelco, Bellefonte, PA, USA) was exposed to the headspace of the vial for 30 min at 60 °C, then desorbed into the injector port in 3 min at 250 °C. Extracted volatile compounds were analyzed by gas chromatography-mass spectrometry (GC-MS) (TSQ Quantum XLS, Thermo Fisher Scientific, Waltham, MA, USA). Volatile constituents were identified through mass spectral matching against the NIST 2014 and Wiley 11 databases. Only compounds with both similarity index (SI) and reverse similarity index (RSI) above 800 were retained for subsequent data evaluation. Semi-quantification was performed by comparing the peak area of each volatile compound against the peak area of the internal standard. The final results were expressed as 2,4,6-trimethylpyridine equivalents (μg/kg). The dataset was analyzed on the Majorbio Cloud Platform. Principal component analysis (PCA) was employed, and a heatmap was generated to display the profiles of the detected volatile compounds among different treated groups.

### 2.6. Metabolite Analysis

#### 2.6.1. Extraction of Metabolites

For metabolite extraction, 100 mg of muscle tissue was homogenized in a 2 mL centrifuge tube with a 6 mm grinding bead, using 800 μL of extraction solution (methanol: water = 4:1, *v*/*v*) containing internal standards. Homogenization was performed with a Wonbio-96c frozen tissue grinder (Shanghai Wanbo Biotechnology Co., Ltd., Shanghai, China) at −10 °C and 50 Hz for 6 min, followed by ultrasonic extraction at 5 °C and 40 kHz for 30 min. After incubation at −20 °C for 30 min, the samples were centrifuged at 13,000 g for 15 min at 4 °C. The resulting supernatant was collected for further analysis.

#### 2.6.2. UHPLC-MS/MS Analysis

The metabolomic profiling was carried out by LC-MS/MS using a UHPLC-Q Exactive HF-X system. Separation was performed on an ACQUITY HSS T3 column (100 mm × 2.1 mm, 1.8 μm) with the column temperature set at 40 °C. A binary mobile phase system of 0.1% formic acid in water: acetonitrile (2:98, *v*/*v*, A) and 0.1% formic acid in acetonitrile (B) was used at a flow rate of 0.40 mL/min, with an injection volume of 5 μL. Mass spectrometry detection was performed using an ESI source in both positive and negative polarity modes. The source temperature was maintained at 400 °C, with sheath and auxiliary gas flows set to 40 and 10 arb, respectively. The spray voltages were set to 3500 V (positive) and −2800 V (negative). Full MS scans (70–1050 *m*/*z*) were followed by data-dependent MS/MS scans with normalized collision energies of 20, 40, and 60 V. A pooled quality control (QC) sample was prepared by mixing equal aliquots from all samples to monitor system stability and instrument performance.

#### 2.6.3. Data Processing

The raw data were preprocessed using Progenesis QI software (version 3.0, Waters Corporation, Milford, MA, USA). Metabolite identification was carried out through database searching, primarily referencing the Human Metabolome Database (HMDB, link 2), METLIN (link 3), and the self-compiled MJDB database provided by Majorbio Biotechnology Co., Ltd. (Shanghai, China). The resulting data matrix was subsequently uploaded to the Majorbio Cloud Platform (https://cloud.majorbio.com) for further analysis. Missing values were filtered using the 80% rule (retaining variables present in >80% of samples in at least one group) and imputed with the raw minimum value. To minimize variations from sample preparation and instrument instability, sum normalization (total ion current) was applied to the response intensities. Variables with a relative standard deviation (RSD) > 30% in QC samples were excluded, and the remaining data were log10-transformed for subsequent analysis. Multivariate statistical analysis was performed using the ropls package (Version 1.6.2) in R. To exclude model overfitting, permutation testing with 200 permutations was conducted. Differential metabolites were defined with thresholds of VIP > 1 and *p* < 0.05. Hierarchical clustering analysis was performed using Euclidean distance to assess correlations and distances among metabolites, with results visualized as a triangular heatmap. Differential metabolites between groups were subjected to metabolic enrichment and pathway analysis based on the Kyoto Encyclopedia of Genes and Genomes (KEGG) pathway database (http://www.genome.jp/kegg/, accessed on 12 December 2025), facilitating the mapping of key metabolites onto relevant biochemical pathways. The samples were labeled as Control_ (0 mg/kg) and H_Se (2.0 mg/kg) according to the platform’s pipeline.

### 2.7. Statistical Analysis

All data are expressed as mean ± standard deviation (SD) based on cage-level replicates. Statistical analyses were conducted on cage-level means (n = 3 cages per treatment) using SPSS 19.0 (version 22.0; IBM Corp., New York, NY, USA), with the net cage serving as the experimental unit. Prior to analysis, data normality was assessed using the Shapiro–Wilk test, and homogeneity of variances was verified using Levene’s test. One-way analysis of variance (ANOVA) followed by Duncan’s multiple-range test was used to evaluate significant differences among groups (*p* < 0.05). The complete statistical results, including degrees of freedom (df), F-values, and exact *p*-values for all parameters, are provided in the Supplementary Material (Table S5). To estimate the optimal dietary selenium concentration, a second-order polynomial regression analysis was performed based on the ICP-MS-measured feed selenium concentrations. The Majorbio Cloud Platform (https://cloud.majorbio.com) was used for all bioinformatics analyses.

## 3. Results and Discussion

### 3.1. Determination of Biochemical Properties

#### 3.1.1. Determination of Growth Performance

During the 8-week feeding trial, 100% survival was maintained in all dietary treatments, confirming that sodium selenite supplemented at graded levels (0.0–2.0 mg Se kg^−1^ diet) exerted no mortality on adult *Cyprinus carpio*. Growth performance indices are presented in Table S2. No significant differences (*p* > 0.05) were detected in condition factor (CF), visceral somatic index (VSI), or hepatosomatic index (HSI) across all groups. Weight gain rate (WGR) exhibited a quadratic dose–response pattern, increasing incrementally from 42.29 ± 2.27% (control) to a peak of 53.87 ± 0.73% at 1.0 mg Se kg^−1^, followed by a slight reduction to 50.03 ± 2.23% at 2.0 mg Se kg^−1^. A similar trend was observed for the specific growth rate (SGR), which was significantly higher in all selenium-supplemented groups (0.70 ± 0.01%, 0.72 ± 0.01%, and 0.68 ± 0.02% for 0.5, 1.0, and 2.0 mg/kg, respectively) than in the control group (0.58 ± 0.03%) (*p* < 0.05). Based on the quadratic regression analysis (Figure S1), the relationship between dietary selenium levels and growth performance was significant. The regression model for WGR yielded the equation Y= −8.501X^2^ + 30.672X + 26.915 (R^2^ = 0.908, *p* < 0.001), estimating the optimal dietary selenium level at 1.804 mg/kg with a maximum WGR of 54.58%. Similarly, for SGR, the model (Y= −0.096X^2^ + 0.345X + 0.415, R^2^ = 0.905, *p* < 0.001) indicated an optimal selenium requirement of 1.797 mg/kg to achieve a maximum SGR of 0.725%/day.

These results demonstrate that dietary sodium selenite significantly enhances growth performance in adult common carp, consistent with reports demonstrating growth-promoting effects of Se in grass carp (*Ctenopharyngodon idellus*) [22]. Notably, the optimal sodium selenite requirement determined herein (1.797–1.804 mg/kg) is higher than that reported for juvenile blunt snout bream (*Megalobrama amblycephala*; 0.958–0.959 mg/kg) [23], lower than that for juvenile Nile tilapia (*Oreochromis niloticus*; 2.0 mg/kg) [24]. Such interspecies variations are likely attributable to differences in species physiology, life stage, and dietary matrix composition [25], reinforcing the need for stage- and species-specific Se optimization in finfish feeds.

#### 3.1.2. Determination of Proximate Composition

The effects of dietary sodium selenite on muscle proximate composition are illustrated in Figure S2. Moisture content did not differ significantly among treatments. Ash content exhibited a slight increase from 1.11% in the control group to 1.12%, 1.15%, and 1.17% in the 0.5, 1.0, and 2.0 mg/kg Se groups, respectively. Conversely, crude fat content declined from 9.64% in the control group to 9.54%, 9.12%, and 9.03% in the corresponding selenium-supplemented groups. Despite these trends, neither ash nor crude fat content differed significantly across selenium levels.

Notably, crude protein content increased significantly (*p* < 0.05) in a dose-dependent manner, rising from 19.02% (control) to 19.20, 19.56, and 19.60% in the 0.5, 1.0, and 2.0 mg Se kg^−1^ groups, respectively. These data suggest that dietary sodium selenite enhances muscle protein deposition in common carp. This effect may be related to the critical role of Se in selenoprotein synthesis, which supports nitrogen retention and protein anabolism, thereby contributing to improved growth performance [26]. Comparable increases in muscle protein have been documented in Nile tilapia (*Oreochromis niloticus*) fingerlings [27] and rainbow trout [28]. However, other studies report no significant effects of Se on proximate composition [16,29], discrepancies that may arise from variations in fish species, developmental stage, diet formulation, or Se source [30].

#### 3.1.3. Determination of Antioxidant Enzyme Activity

The impacts of dietary sodium selenite on antioxidant biomarkers in muscle and liver are summarized in Figure 1. In muscle, glutathione peroxidase (GSH-Px) activity increased dramatically in a dose-dependent manner, rising from 1.24 ± 0.03 U/mg prot in the control to 9.16 ± 0.24, 11.65 ± 0.94, and 12.97 ± 1.52 U/mg prot at 0.5, 1.0, and 2.0 mg/kg Se, respectively. Catalase (CAT) activity exhibited a similar trend, reaching 1.50-, 2.39-, and 2.83-fold that of the control. Superoxide dismutase (SOD) activity increased significantly from 9.04 ± 1.19 to 13.06 ± 0.05, 13.25 ±0.25, and 15.18 ± 2.28 U/mgprot (*p* < 0.05), although no significant differences were detected among the selenium-supplemented groups. Malondialdehyde (MDA), a marker of lipid peroxidation, decreased significantly (*p* < 0.05) from 3.92 ± 0.39 to 2.61 ± 0.19, 1.54 ± 0.25, and 1.47 ± 0.25 nmol/mg protein with increasing dietary Se levels. Overall, Se supplementation markedly improved the antioxidant capacity of muscle, with no significant differences observed in GSH-Px, SOD, and MDA content between the 1.0 and 2.0 mg Se kg^−1^ groups.

In the liver, GSH-Px activity increased from 105.48 ± 3.32 (control) to 208.82 ± 10.89, 227.52 ± 12.50, and 234.29 ± 3.21 U/mg prot as dietary selenium increased. Meanwhile, hepatic MDA content decreased from 1.35 ± 0.11 to 1.12 ± 0.09, 1.02 ± 0.07, and 0.96 ± 0.02 mmol/mg prot. No significant differences in GSH-Px or MDA were found between the 1.0 and 2.0 mg Se kg^−1^ selenium-supplemented groups (*p* < 0.05). CAT activity was significantly higher in the 2.0 mg Se kg^−1^ group (61.85 ± 1.25 mg prot/mL) relative to the control (53.03 ± 2.38 mg prot/mL). By contrast, hepatic SOD activity did not show significant alterations across all experimental groups.

Selenium is an essential cofactor for GSH-Px, in which it is incorporated as selenocysteine at the active site. Together with SOD and CAT, GSH-Px forms the primary enzymatic antioxidant barrier against reactive oxygen species (ROS) [31]. The observed increases in GSH-Px, CAT, and SOD activities, coupled with reduced MDA levels, suggest that dietary sodium selenite effectively augments antioxidant defense systems and alleviates oxidative stress in both muscle and liver of common carp. These results align with previous studies demonstrating that Se supplementation upregulates antioxidant enzyme expression and reduces lipid peroxidation in finfish [22,28].

### 3.2. Determination of Selenium Content, Speciation, and Heavy Metal Antagonism

#### 3.2.1. Determination of Selenium Concentration

Tissue Se concentrations in muscle and liver of common carp are presented in Figure 2A. Se concentration increased in a dose-dependent manner in both tissues with increasing dietary sodium selenite supplementation. In muscle, Se levels rose significantly from 189.44 ± 14.83 ppb (control) to 311.33 ± 42.90 ppb and 416.11 ± 95.28 ppb at 1.0 and 2.0 mg Se kg^−1^, respectively. A marked elevation in muscle Se deposition was observed above 1.0 mg/kg, indicating effective Se assimilation and retention in edible tissue.

Hepatic Se concentrations were consistently and markedly higher than those in muscle, reaching 578.58 ± 96.99, 897.31 ± 87.94, 1005.59 ± 19.05, and 1421.79 ± 78.49 ppb with increasing dietary Se. Hepatic Se levels were approximately threefold higher than in muscle, in agreement with observations in juvenile common carp [32]. This may be attributed to the liver’s role in mineral metabolism, detoxification, and nutrient storage, which favors Se sequestration relative to peripheral tissues [33].

The dose-dependent Se deposition observed herein is consistent with reports in *Paramisgurnus dabryanus* [34] and blunt snout bream (*Megalobrama amblycephala*) [23], and correlates with the modest elevation in muscle ash content (Figure S2B). Importantly, Se-enriched common carp muscle may represent a valuable dietary Se source for human consumption, supporting the potential application of Se-fortified feeds to improve the nutritional quality of aquaculture products.

#### 3.2.2. Determination of Heavy Metal Antagonism

The antagonistic effects of dietary Se on muscle mercury (Hg) and cadmium (Cd) accumulation are illustrated in Figure 2B. Strong inverse relationships were observed between dietary Se levels and heavy metal concentrations. Supplementation with 2.0 mg Se kg^−1^ significantly (*p* < 0.05) reduced muscle Hg from 3.91 ± 0.65 to 1.60 ± 0.46 ppb and Cd from 0.43 ± 0.08 to 0.12 ± 0.02 ppb relative to the control.

These findings support the detoxification potential of Se against co-occurring heavy metals in farmed fish. A similar reduction in Cd levels was reported in the muscle of grass carp (*Ctenopharyngodon idella*) fed sodium selenite, although no significant decrease in Hg was observed in that study [35]. In contrast, the detoxifying effect of Se against Hg has been confirmed in rainbow trout [36]. The observed reduction in tissue heavy metal burden suggests a potential antagonistic interaction. It has been hypothesized in the previous literature that this protective effect may involve the formation of insoluble, inert Se-metal complexes (such as Hg-Se or Cd-Se) that facilitate excretion rather than tissue deposition [37].

In addition, as the catalytic core of GSH-Px, Se enhances the enzyme’s activity (Figure 1), which likely contributes to the neutralization of reactive oxygen species generated by heavy metal exposure, thereby reducing oxidative injury [38]. Collectively, these results indicate that dietary sodium selenite mitigates Hg and Cd bioaccumulation while improving antioxidant status, representing a promising strategy to enhance food safety and quality in common carp aquaculture.

### 3.3. Determination of Flesh Quality and Water-Holding Capacity

The effects of dietary sodium selenite supplementation on textural properties and water-holding capacity (WHC) of common carp muscle are presented in Figure 3. Hardness was significantly elevated (*p* < 0.05) in the 1.0 and 2.0 mg Se kg^−1^ groups, reaching 582.32 ± 64.83 g and 605.94 ± 76.50 g, respectively, compared with 452.13 ± 31.30 g in the control group. Springiness was significantly improved in all Se-supplemented treatments (*p* < 0.05), with no significant differences observed among graded Se levels. Gumminess and chewiness followed similar patterns: values increased significantly from 0.5 to 1.0 mg Se kg^−1^ and declined slightly at 2.0 mg Se kg^−1^. Resilience also increased in a dose-dependent manner, rising from 0.22 ± 0.04 (control) to 0.34 ± 0.03, 0.35 ± 0.04, and 0.42 ± 0.09 at 0.5, 1.0, and 2.0 mg Se kg^−1^, respectively.

Texture profile analysis (TPA) parameters reflect the mechanical properties of muscle tissue and directly influence fish quality. The observed improvements in hardness, springiness, gumminess, chewiness, and resilience align with the physiological responses observed in largemouth bass (*Micropterus salmoides*) fed Se-supplemented diets [39]. To our knowledge, specific data regarding the effects of selenium on the textural properties of common carp are limited. Elevated oxidative stress can induce the cross-linking and degradation of myofibrillar proteins, ultimately resulting in muscle softening and a loss of elasticity. It is plausible that by scavenging ROS and suppressing lipid peroxidation (as presented in Figure 1), sodium selenite helps to maintain the structural integrity of myofibrils and cell membranes, which may partly explain the enhanced textural properties observed in this study [40].

In addition to texture, centrifugal loss (Figure 3F) was significantly reduced (*p* < 0.05) in all Se-supplemented groups relative to the control (6.49 ± 1.52%), decreasing to 2.97 ± 1.03%, 3.02 ± 0.45%, and 4.41 ± 0.72% with increasing dietary Se. While direct comparisons in adult carp are scarce, similar improvements in WHC have been documented in blunt snout bream (*Megalobrama amblycephala*) fed dietary selenium [41]. The marked reduction in centrifugal loss in the present study indicates enhanced water-holding capacity, which was consistent with the improved textural properties. The accumulation of ROS can trigger the oxidation of myofibrillar proteins and might disrupt their three-dimensional hydrated network, leading to increased water loss. Excessive water loss is often accompanied by soluble protein leakage, leading to inferior texture, dry mouthfeel, and reduced muscle integrity [42]. In our study, sodium selenite may reduce water loss by protecting myofibrillar proteins from ROS-induced oxidative damage, thus maintaining their structural integrity and water-binding capacity.

Collectively, these results indicate that dietary sodium selenite supplementation at 1.0–2.0 mg/kg significantly improves textural properties and water retention in common carp muscle, thereby enhancing overall flesh quality. Notably, while organic Se sources generally exhibit higher bioavailability, sodium selenite has been reported to improve flesh quality more effectively than Se-yeast or Se-enriched *Spirulina platensis* in yellow catfish (*Pelteobagrus fulvidraco*) [43]. The beneficial effects observed in this study are likely attributable to the enhancement of antioxidant capacity and the reduction in lipid peroxidation, as supported by the measured redox indicators (Figure 1).

### 3.4. Determination of Nutritional, Taste and Volatile Flavor Analysis

#### 3.4.1. Determination of Hydrolyzed Amino Acid Composition

Amino acids serve as the primary structural components of muscle proteins and are critical determinants of both the nutritional value and taste profile of aquatic products. The effects of graded dietary sodium selenite on hydrolyzed amino acid profiles in common carp muscle are summarized in Table S3. A total of 17 amino acids were quantified following acid hydrolysis, including aspartic acid (Asp), glutamic acid (Glu), glycine (Gly), alanine (Ala), threonine (Thr), serine (Ser), proline (Pro), arginine (Arg), histidine (His), phenylalanine (Phe), tyrosine (Tyr), cysteine (Cys), valine (Val), methionine (Met), lysine (Lys), isoleucine (Ile), and leucine (Leu). It should be noted that cysteine is susceptible to oxidative degradation under the hydrolysis condition without a performic acid pre-oxidation step, and thus Cys data were excluded. Although nitrogen-blanketing mitigated oxidative damage, partial oxidation of Met may still take place, so methionine values are retained but should be regarded as approximate.

Total amino acid (TAA) content exhibited a slight increasing trend, rising from 16.56 ± 0.65 g/100 g in the control group to 17.32 ± 0.85 and 17.28 ± 1.11 g/100 g in the 1.0 and 2.0 mg Se kg^−1^ groups, respectively. This numerical elevation was consistent with the concomitant increase in crude protein content observed in response to dietary Se supplementation (Figure S2D). However, no significant differences (*p* > 0.05) were detected among treatments in terms of TAA, total essential amino acids (EAA), non-essential amino acids (NEAA), or the EAA/NEAA ratio. These findings indicate that dietary sodium selenite, within the concentration range tested, exerted no significant modulatory effect on the hydrolyzed amino acid composition of common carp muscle. The adequate selenium supplementation in this study likely enhanced the activity of selenoproteins (e.g., GSH-Px), thereby scavenging excess ROS and protecting muscle structural proteins from oxidative degradation. This preservation effect helps maintain the inherent nutritional equilibrium of muscle amino acids while supporting protein deposition. The present results are consistent with previous observations in blunt snout bream fed diets supplemented with different selenium sources [41].

#### 3.4.2. Determination of Fatty Acid Composition

The effects of dietary sodium selenite supplementation on muscle fatty acid profiles of adult common carp are presented in Table 1. Muscle lipids were dominated by unsaturated fatty acids (UFA), which accounted for approximately 80% of total fatty acids, whereas saturated fatty acids (SFA) comprised only about 20%. This profile underscores the high nutritional value of common carp as a health-promoting food for human consumption.

Although SFA content exhibited a slight reduction at 0.5 mg Se kg^−1^ (with a corresponding increase in UFA), no significant differences (*p* > 0.05) were observed among treatments in total monounsaturated fatty acids (MUFA) or polyunsaturated fatty acids (PUFA). Notably, the combined content of eicosapentaenoic acid (EPA) and docosahexaenoic acid (DHA) increased significantly (*p* < 0.05) from 3.55 ± 0.09% in the control group to 4.78 ± 0.39% and 5.14 ± 0.61% in the 1.0 and 2.0 mg Se kg^−1^ groups, respectively. Furthermore, the atherogenic index (AI) displayed a decreasing trend with increasing dietary Se, declining from 1.86 ± 0.04 (control) to 1.53 ± 0.31, 1.47 ± 0.16, and 1.47 ± 0.55 at 0.5, 1.0, and 2.0 mg Se kg^−1^, respectively; however, these differences were not statistically significant (*p* > 0.05).

Dietary fatty acids represent critical nutrients that regulate physiological function and human health outcomes. High concentrations of n − 3 PUFAs, especially EPA and DHA, are widely recognized for their cardioprotective effects and ability to reduce the risk of chronic metabolic diseases [44]. Consistent with the present findings, dietary Se supplementation has been reported to enhance EPA and DHA deposition in a range of farmed finfish, including grass carp (*Ctenopharyngodon idella*) [35], *Onychostoma macrolepis* [45], and *Epinephelus coioides* [46]. The significant accumulation of EPA and DHA in the present study is closely linked to the antioxidant properties of selenium. PUFAs are highly susceptible to ROS-induced lipid peroxidation, which can lead to fatty acid degradation and reduced deposition [47]. Sodium selenite activates selenoproteins to scavenge ROS and suppress peroxidation, thereby preserving PUFA structural integrity and facilitating their stable deposition in muscle. The observed EPA + DHA increase is corroborated by our redox indicators (Figure 1), where Se supplementation was associated with reduced MDA and elevated GSH-Px, CAT, and SOD activities.

The atherogenic index (AI) serves as a key indicator for evaluating the cardioprotective potential of dietary lipids, with lower values reflecting reduced cardiovascular risk. In the present study, the downward trend in AI, coupled with the significant elevation in n − 3 EPA + DHA content, demonstrates that sodium selenite supplementation is associated with an enhancement of the lipid nutritional quality of common carp muscle. These results support the potential utility of Se-fortified feeds to produce value-added aquaculture products with enhanced health-promoting properties for human consumption.

#### 3.4.3. Determination of Free Amino Acid Composition

The effects of dietary sodium selenite on free amino acid (FAA) profiles in common carp muscle are presented in Table 2. Similar to hydrolyzed amino acid analysis, free cysteine was undetectable due to oxidation during sample preparation, whereas methionine was quantified with minor oxidation interference. Total free amino acid (TFAA) content exhibited a slight initial decrease from 564.56 ± 21.75 mg/100 g (control) to 556.19 ± 37.37 mg/100 g at 0.5 mg Se kg^−1^, followed by a gradual increase to 566.92 ± 7.67 and 584.10 ± 29.51 mg/100 g at 1.0 and 2.0 mg Se kg^−1^, respectively. However, no significant differences (*p* > 0.05) were observed among treatments in TFAA or total bitter amino acids (BAAs).

It is noteworthy that total umami amino acids (UAAs) increased significantly (*p* < 0.05) from 17.62 ± 4.28 mg/100 g in the control to 27.09 ± 2.44 and 25.89 ± 1.88 mg/100 g in the 1.0 and 2.0 mg Se kg^−1^ groups, respectively. Glu content, the primary umami contributor, was elevated by 1.36- to 1.65-fold in Se-supplemented groups relative to the control. Similarly, total sweet amino acids (SAAs) were significantly higher (*p* < 0.05) at 2.0 mg Se kg^−1^ (213.70 ± 8.25 mg/100 g) compared with the control (199.53 ± 4.75 mg/100 g), with Gly showing a 1.19-fold increase.

FAAs are well-established determinants of taste perception and precursors for volatile aroma generation [48]. His, Gly, and Pro were the most abundant FAAs in common carp muscle across all treatments. The significant elevation in UAAs, especially Glu, likely contributes to enhanced umami intensity, particularly through synergistic interactions with 5′-inosine monophosphate (IMP) and 5′-adenosine monophosphate (AMP) [49]. Furthermore, the marked increase in SAAs (notably Gly) at 2.0 mg Se kg^−1^ may further improve overall palatability, as Gly and Glu are known to synergistically enhance umami and sweet notes in aquatic products [50]. Collectively, these results suggest that dietary sodium selenite supplementation enhances the accumulation of taste-active FAAs, particularly umami- and sweet-enhancing components.

#### 3.4.4. Determination of Volatile Compounds

The categories and relative contents of volatile compounds are summarized in Table S4, while the corresponding percentage composition is illustrated in Figure 4A. Principal component analysis (PCA) of the flavor compound composition across different samples was presented in Figure 4B. It revealed that PC1 accounted for 94.93% of the variance, whereas PC2 explained 4.30%. The cumulative variance contribution of these two components reached 99.23%. Samples fed with different concentrations of dietary selenium were clearly distinguished, indicating good reproducibility among replicates of the same treatment. To visually classify and compare the effects of dietary selenium levels on volatile compounds in fish muscle, the volatile flavor components obtained from SPME-GC-MS analysis were visualized for intuitive data comparison (Figure 4C).

The results indicated that a total of 40 volatile compounds were identified in the muscle of fish fed diets with different selenium concentrations, including 8 aldehydes, 8 alcohols, 8 aromatic compounds, 6 esters, 4 ketones, 3 acids, 2 alkanes, and 1 amine. The total volatile content, expressed as 2,4,6-trimethylpyridine equivalents, increased from 5231.93 ± 125.49 μg/kg in the control group to 7065.79 ± 346.34 μg/kg, 9722.70 ± 838.48 μg/kg, and 10,794.18 ± 1018.85 μg/kg in the 0.5, 1.0, and 2.0 mg/kg Se-supplemented groups, respectively. The results indicated that dietary selenium supplementation enhanced the flavor profile of the muscle in a dose-dependent manner.

Aldehydes were the most abundant volatile components in fish muscle, confirming that aldehydes are the primary contributors to the volatile odor profile, which was consistent with results obtained in the muscle of large yellow croaker *Larimichthys crocea* [51]. Among the individual aldehydes, hexanal was the most abundant. Its percentage decreased markedly at 0.5 mg/kg of dietary selenium, but increased significantly at levels of 1.0 and 2.0 mg/kg. Hexanal has been reported to play a dual role in fish aroma: it contributes to the fresh fish aroma in fresh samples, while during processing its excessive accumulation is a classic marker of lipid oxidative rancidity, responsible for off-flavors in fish muscle [52]. The significant accumulation of hexanal in the high-Se groups (1.0 and 2.0 mg/kg) can be explained by the substrate-driven oxidation hypothesis. Although sodium selenite enhances antioxidant enzyme activities, which protect PUFAs from degradation and promote the deposition of EPA and DHA, the substantially elevated PUFA pool provides a greater abundance of oxidizable substrates. Consequently, even with stronger antioxidant defenses, the absolute formation of aldehydes (hexanal) may increase due to this higher substrate availability. Furthermore, at high-Se groups, the antioxidant system may approach saturation, or selenium may exert a pro-oxidant effect, leading to insufficient protection against lipid oxidation. Therefore, while the 1.0–2.0 mg/kg Se dose range is optimal for maximizing growth, it inadvertently compromises sensory quality due to the accumulation of rancid off-flavors. Future practical applications should carefully consider this trade-off [39]. Nonanal, octanal, and other straight-chain saturated aldehydes, which are recognized as key components of fresh fish aroma, showed a consistent increasing trend with dietary Se supplementation up to 1.0 mg/kg, followed by a moderate decline at 2.0 mg/kg. Unsaturated aldehydes like (E)-2-octenal and (E, E)-2, 4-nonadienal, associated with green, fatty, and citrus flavors, peaked at 1.0 mg/kg Se. Their levels declined slightly at 2.0 mg/kg, but remained significantly higher than the control. Aldehydes are primarily derived from unsaturated fatty acid hydroperoxides through the action of lipoxygenase [53].

The total alcohol content exhibited a trend of first increasing and then decreasing, with 1-hexanol and 1-pentanol being the primary contributors to the total alcohol content. Specifically, their contents in the 1.0 mg/kg Se group were 2.15-fold and 4.48-fold higher than those in the control group, respectively. 1-Hexanol imparts a floral and fatty odor, while 1-pentanol has a pungent alcoholic odor. Additionally, 1-penten-3-ol, which is characterized by a mushroom-like flavor, also increased significantly with sodium selenite supplementation, peaking in the 1.0 mg/kg Se group. It is formed from the degradation of linoleic acid hydroperoxide [54]. In contrast, phenylethyl alcohol, which possesses a rose-like flavor, showed a decreasing trend similar to that of benzaldehyde. This reduction in phenylethyl alcohol content may be associated with the consumption of phenylalanine during the synthesis of other flavor-related substances. As the alcohols are derived from the oxidation of unsaturated fatty acids, their elevated levels suggest that sodium selenite supplementation may enhance the formation of floral and fatty aromas in common carp muscle [55].

Esters, predominantly synthesized via the esterification of alcohols and fatty acids, are key contributors to fruity, floral, and sweet flavor in fish [56]. The main components, methyl tetradecanoate and methyl hexadecanoate, have odor description of fatty, balsamic, honey, orris, and green, fruity and fatty increased by 3.54–4.55-fold and 3.17–4.39-fold, respectively. These long-chain fatty acid methyl esters likely contribute fruity and waxy notes, thereby enhancing the overall flavor quality. Ketones primarily originate from the degradation of unsaturated fatty acids, degradation of amino acids, Maillard reactions, or microbial oxidation, contributing distinct odors [57]. They typically impart weak, specific odors. The increase in the content of (E, E)-3, 5-octadien-2-one indicated that sodium selenite may intensify the animal grease flavor, potentially modulating the overall fish aroma [58].

### 3.5. Determination of Untargeted Metabolomics

In the present study, untargeted metabolomics was initially performed between the control and the 2.0 mg/kg sodium selenite group (H_Se). This highest supplementation level elicited the most pronounced phenotypic alterations, including significant improvements in growth performance, selenium accumulation, flesh quality, and volatile profiles. We prioritized this pairwise comparison to capture the major global metabolic remodeling triggered by dietary selenite exposure, serving as a first-line exploratory screening to identify key perturbed pathways.

The partial least squares discriminant analysis (PLS-DA) score plot (Figure 5A) revealed a clear separation between the H_Se and Control_ groups along Component 1 (16.8%), with non-overlapping 95% confidence ellipses. The permutation test was performed to evaluate the validity of the PLS-DA model and assess overfitting. As shown in Figure S3, based on 200 permutations, the original model yielded a high goodness of fit (R^2^ = 0.9689) and a moderate predictive ability (Q^2^ = 0.2297). The permutation test yielded a Q^2^ intercept of 0.2297. Although the positive intercept suggests a potential risk of overfitting, which may arise from the limited sample size or complex biological variation, the regression line exhibits a clear positive slope, and the Q^2^ values from permuted datasets are generally lower than that of the original model. These results suggest that the model’s predictive power is modest [59].

As shown in Figure 5B, a total of 2076 metabolites were commonly detected in both groups, accounting for 92.68% of the total identified metabolites, while 86 (3.84%) and 78 (3.48%) metabolites were uniquely present in the Control_ and H_Se groups, respectively. The high proportion of shared metabolites suggests that selenium supplementation primarily modulates the abundance of core metabolic pathways rather than introducing entirely novel metabolic processes. The superclass annotation of all identified metabolites (Figure 5C) revealed that lipids and lipid-like molecules were the most abundant class (544 metabolites, 34.09%), followed by organic acids and derivatives (331, 20.74%) and organic oxygen compounds (175, 10.96%). It highlights that lipid metabolism is a major target of selenium regulation, which aligns with our previous finding that dietary selenium significantly increased the total EPA and DHA content in muscle by 1.45-fold (Table 1).

The volcano plot (Figure 5D) was used to screen for significantly differential metabolites (SDMs) between the H_Se and Control_ groups, with the criteria of VIP > 1.0 and *p* < 0.05. A total of 222 SDMs were identified, including 137 up-regulated and 85 down-regulated metabolites in the H_Se group. To further visualize the expression patterns, a heatmap of SDMs was constructed in Figure 5E. Notably, the up-regulated subclusters in the H_Se group were highly enriched in n−3 polyunsaturated fatty acids (PUFAs), including docosahexaenoic acid (DHA, 22:6 n−3), Pc (20:5), Lpc (20:5), as well as carnitine derivatives (e.g., linoleoyl carnitine, butyrylcarnitine). This is consistent with the elevated EPA/DHA content observed in our prior study (Table 1), suggesting that selenium may promote the accumulation of n−3 PUFAs in common carp muscle.

KEGG pathway enrichment analysis was performed to explore the biological functions of SDMs. As shown in Figure 5F, the most significantly enriched pathway was Global and overview maps (29 compounds), followed by Lipid metabolism (14 compounds), Amino acid metabolism (9 compounds), Nucleotide metabolism (8 compounds), and Carbohydrate metabolism (7 compounds). Based on the metabolomic pathway enrichment, Figure 5G suggests a potential involvement of pathways such as Biosynthesis of unsaturated fatty acids, Arachidonic acid metabolism, PPAR signaling pathway, and FoxO signaling pathway in the response to selenium supplementation. The PPAR signaling pathway is the core regulatory network for lipid metabolism [60]. Selenium is an essential component of GSH-Px, which protects PUFAs from oxidative degradation [6]. The significantly higher GSH-Px activity in the H_Se group likely reduces lipid peroxidation, thereby preserving and potentially increasing the net content of EPA and DHA in muscle. Furthermore, selenium may up-regulate the expression of fatty acid desaturase and elongase enzymes, which are responsible for the endogenous synthesis of n-3 PUFAs from precursor fatty acids [46]. The increased abundance of carnitine derivatives in the H_Se group also suggests enhanced fatty acid transport and β-oxidation, which supports the efficient utilization and storage of PUFAs in muscle tissue [61]. Additionally, the FoxO signaling pathway, a key regulator of antioxidant gene expression, implies that selenium may enhance the antioxidant capacity of common carp muscle at the molecular level [62].

## 4. Conclusions

In summary, this short-term study suggests that dietary sodium selenite supplementation (0.5–2.0 mg/kg) significantly improves growth performance, muscular antioxidant capacity (elevated GSH-Px, CAT, and SOD, reduced MDA), and water-holding capacity in adult common carp. Doses of 1.0 and 2.0 mg/kg sodium selenite markedly enhanced muscular selenium deposition and exerted a significant antagonistic effect against Hg and Cd bioaccumulation. Additionally, significant increases were observed in hardness, springiness, gumminess, chewiness, resilience, as well as crude protein, umami amino acid content, and the proportional content of EPA + DHA in the same dose treatment group, while higher abundances of aldehyde volatiles were observed. This observation might be attributed to the enlarged PUFA substrate pool available for flavor-related aldehyde formation. Untargeted metabolomic profiling revealed metabolite alterations pointing toward unsaturated fatty acid biosynthesis as well as PPAR- and FoxO-associated metabolic responses, though direct molecular validation of these signaling pathways remains to be conducted.

Collectively, our findings suggest that sodium selenite can serve as a promising feed additive for producing high-quality selenium-enriched common carp, as our results demonstrate that high dietary selenium substantially enhances overall muscle quality despite the concurrent increase in aldehyde levels. However, practical feed formulation should carefully balance these beneficial performance-enhancing effects against adverse flavor-related risks arising from aldehyde accumulation. From a food safety perspective, the muscle selenium concentration in the high-selenium group was 416.11 ± 95.28 μg/kg (0.416 ± 0.095 mg/kg). It should be noted that the historical limit for selenium in fish (1.0 mg/kg) from GB 13105-1992 was canceled. In 2017, China established the daily recommended nutrient intake (RNI) of selenium for adults at 60 μg and the tolerable upper intake level (UL) at 400 μg. Daily consumption of 100 g of fish filet from the high-selenium group would provide approximately 41.6 μg of selenium, accounting for 69% of the RNI and only 10.4% of the UL. Therefore, short-term dietary selenium supplementation at the tested levels is considered safe for human consumption and offers nutritional benefits. Given that this study employed a short-term feeding design mimicking pre-harvest selenium biofortification in adult common carp, our observations regarding heavy metal antagonism and volatile compound profiles cannot be directly extrapolated to long-term aquaculture systems. Further long-term verification and continuous monitoring of tissue residues are warranted.

## Figures and Tables

**Figure 1:**
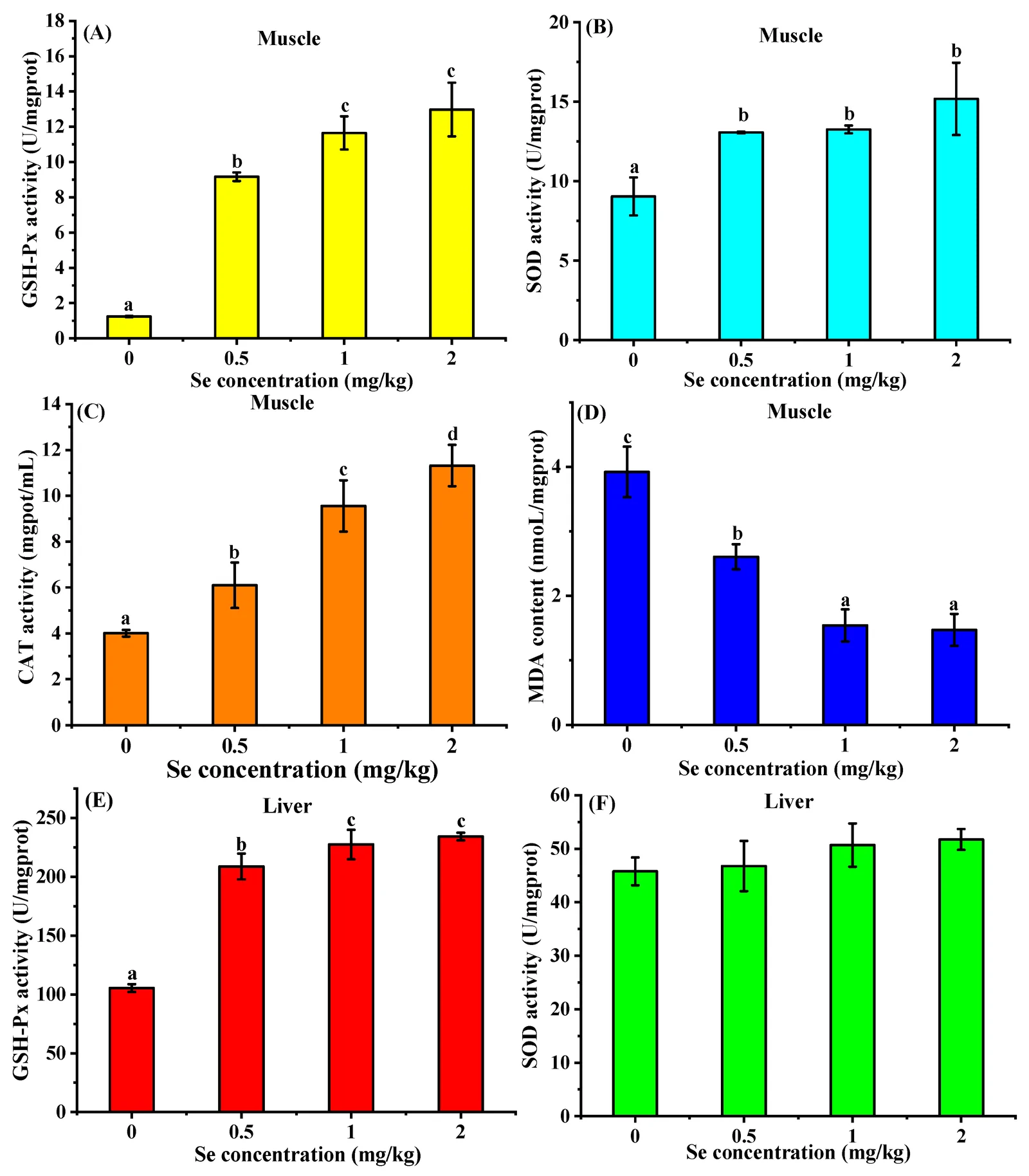

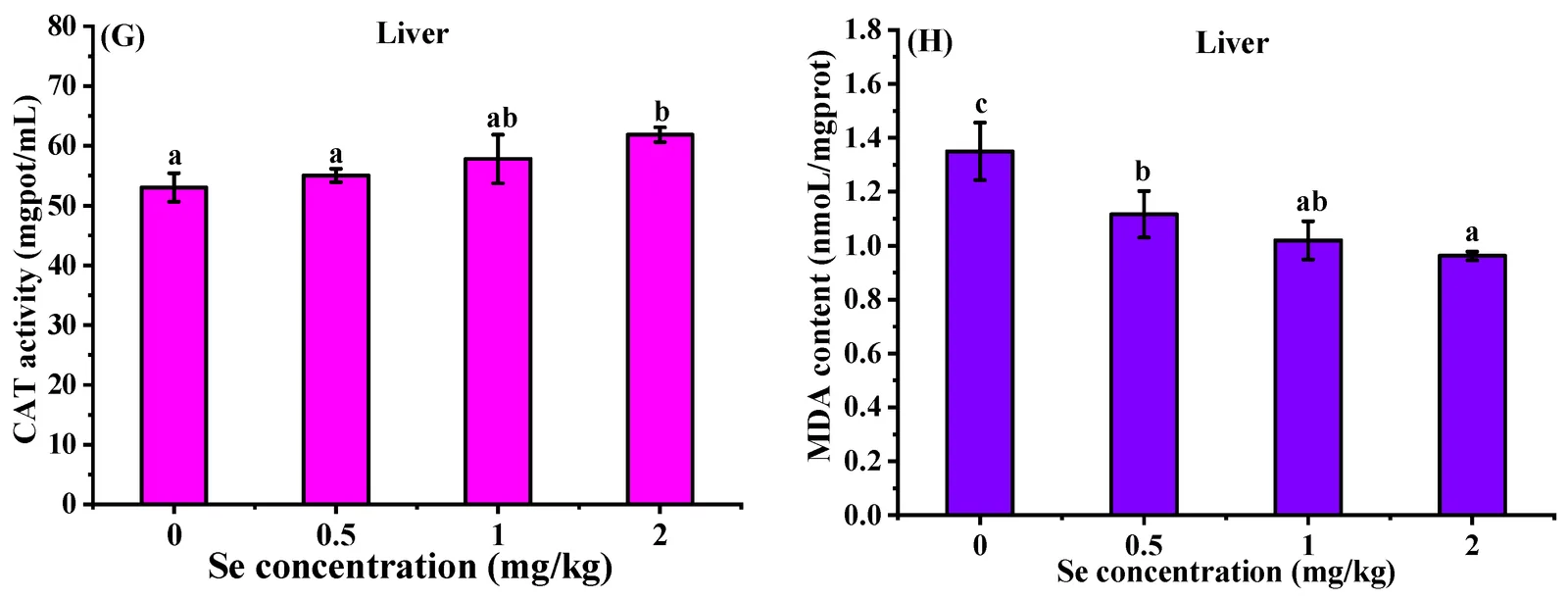
Antioxidant enzyme activities and lipid peroxidation levels in muscle (**A**–**D**) and liver (**E**–**H**) of adult common carp (*Cyprinus carpio*) fed diets supplemented with graded levels of sodium selenite. Data are expressed as means ± SD (*n* = 3 cages per group; 4 fish per cage). Bars with different letters are significantly different (*p* < 0.05).

**Figure 2:**
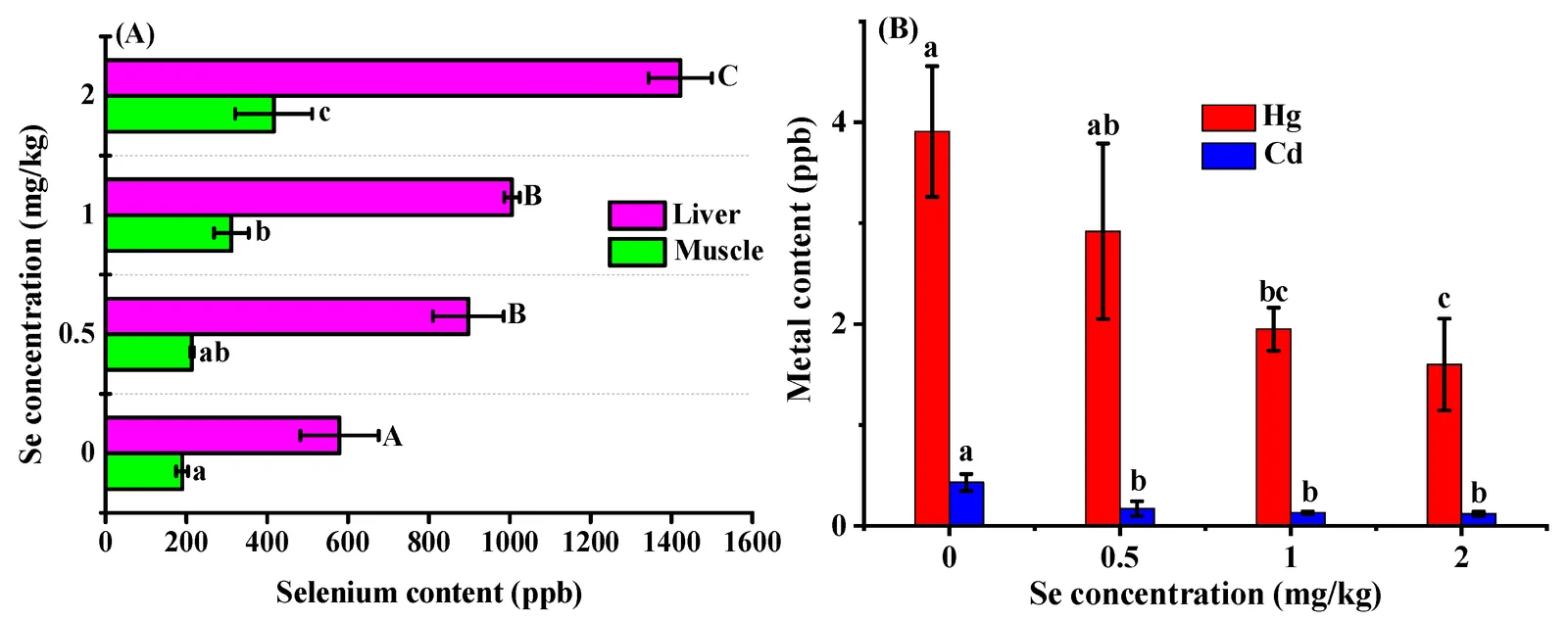
Total selenium concentrations in muscle and liver tissues (**A**). Accumulation of mercury and cadmium in muscle tissue (**B**). Data are expressed as means ± SD (*n* = 3 cages per group). For each cage, tissues from 4 individual fish were pooled to form one composite sample for analytical measurement. Different uppercase letters indicate significant differences in hepatic selenium content, whereas different lowercase letters denote significant differences for muscle selenium content among dietary treatments (*p* < 0.05).

**Figure 3:**
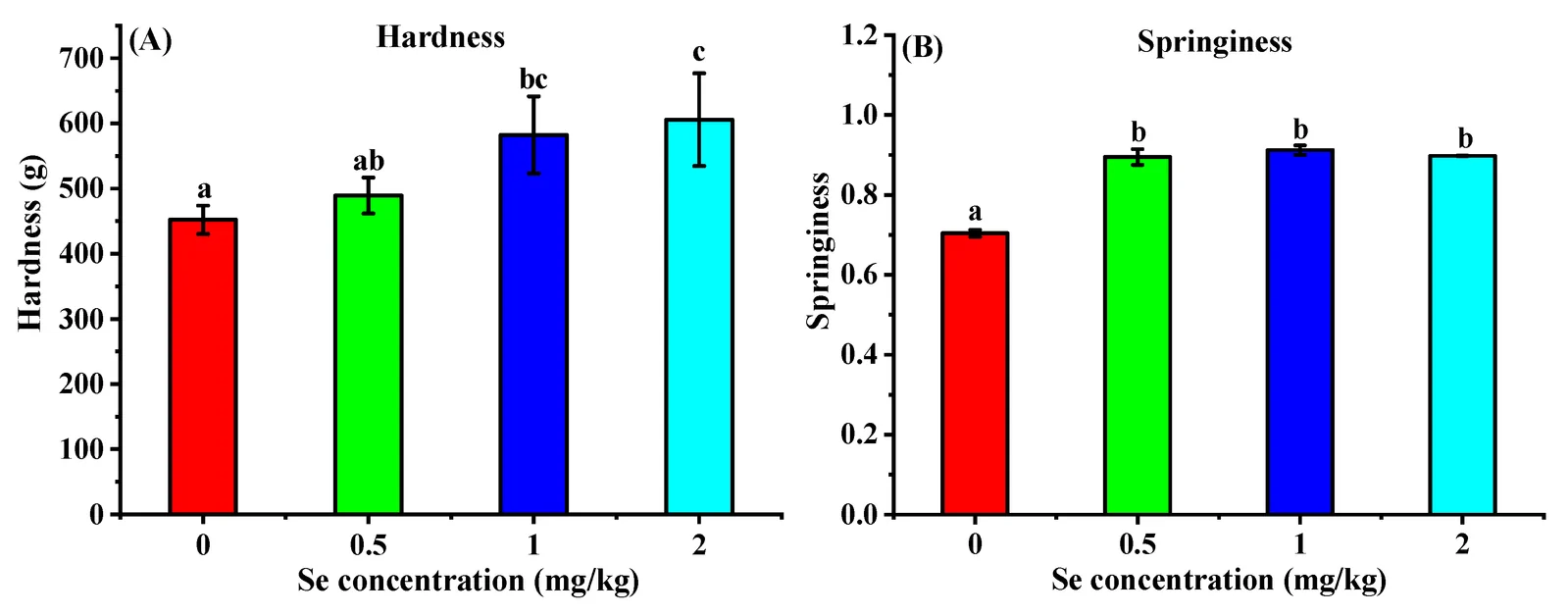

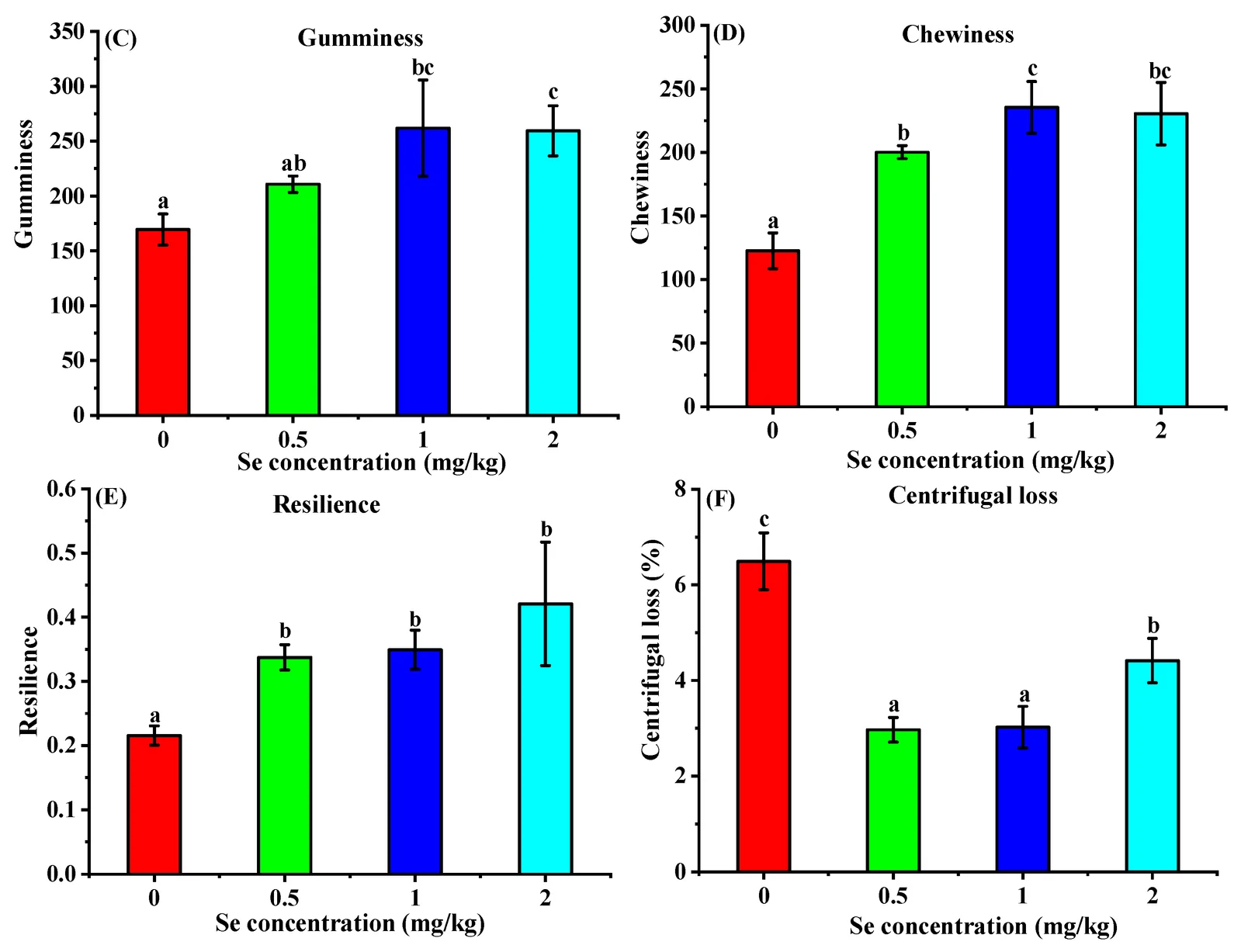
Effects of dietary selenium supplementation on the texture profile-including hardness (**A**), springiness (**B**), gumminess (**C**), chewiness (**D**), and resilience (**E**), as well as centrifugal loss (**F**) in muscle of adult common carp (*Cyprinus carpio*). Data are expressed as means ± SD (*n* = 3 cages per group; 4 fish per cage). Bars with different letters are significantly different (*p* < 0.05).

**Figure 4:**
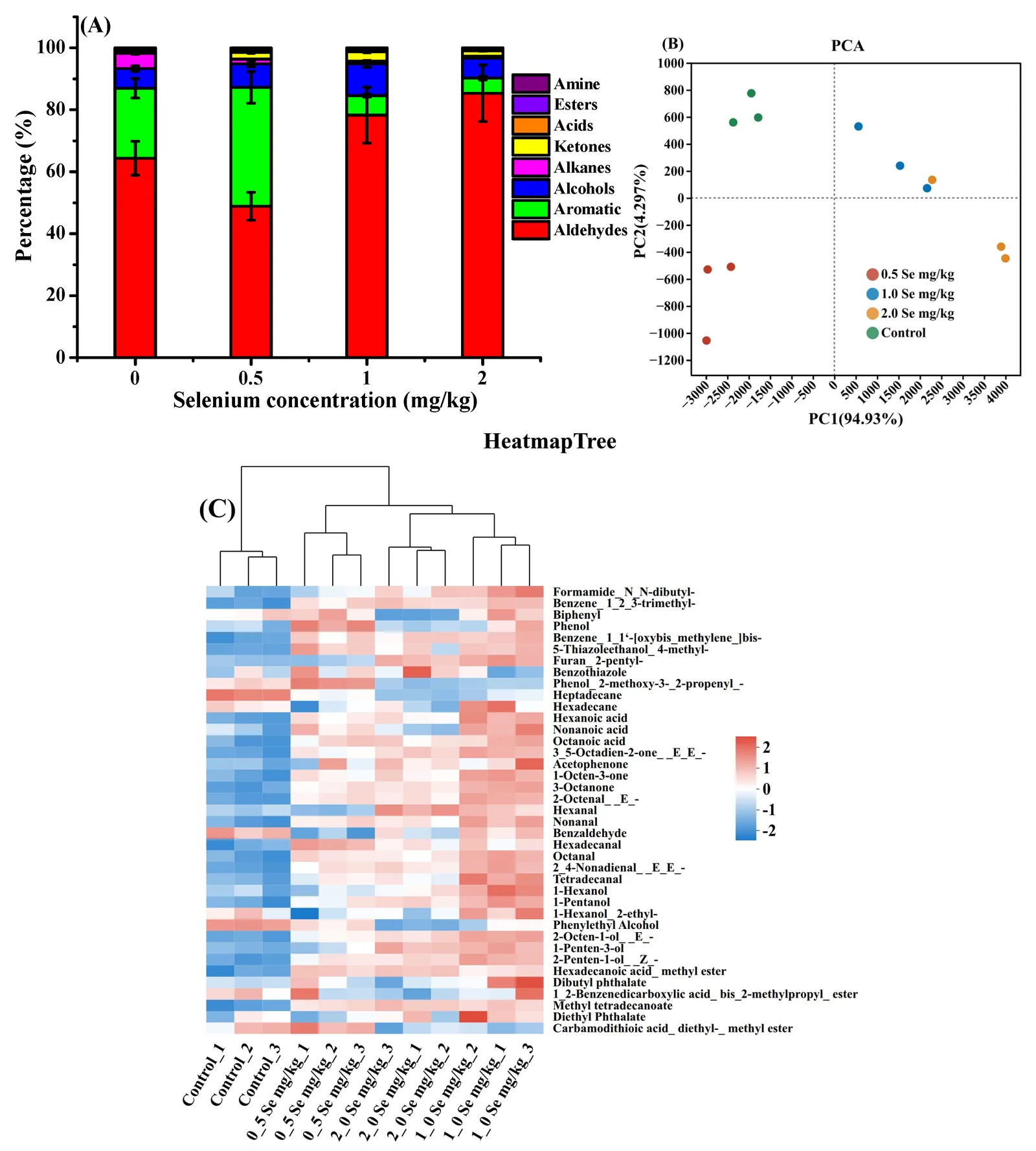
Percentage of volatile compound species (**A**). PCA score plots of the volatile profiles detected by GC-MS (**B**). Heatmap of the volatile compounds of common carp (*Cyprinus carpio*) muscle fed with different concentrations of sodium selenite (**C**). Data are expressed as means ± SD (*n* = 3 cages per group). For each cage, tissues from 4 individual fish were pooled to form one composite sample for analytical measurement.

**Figure 5:**
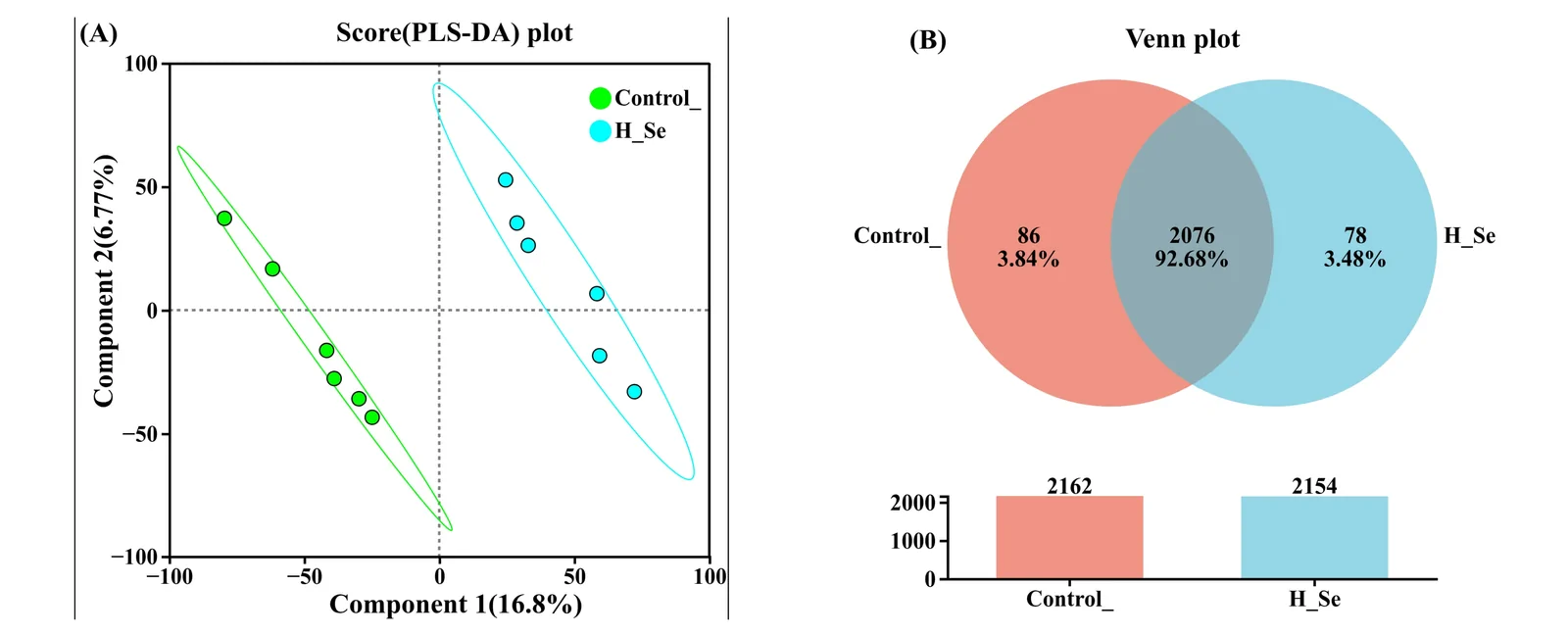

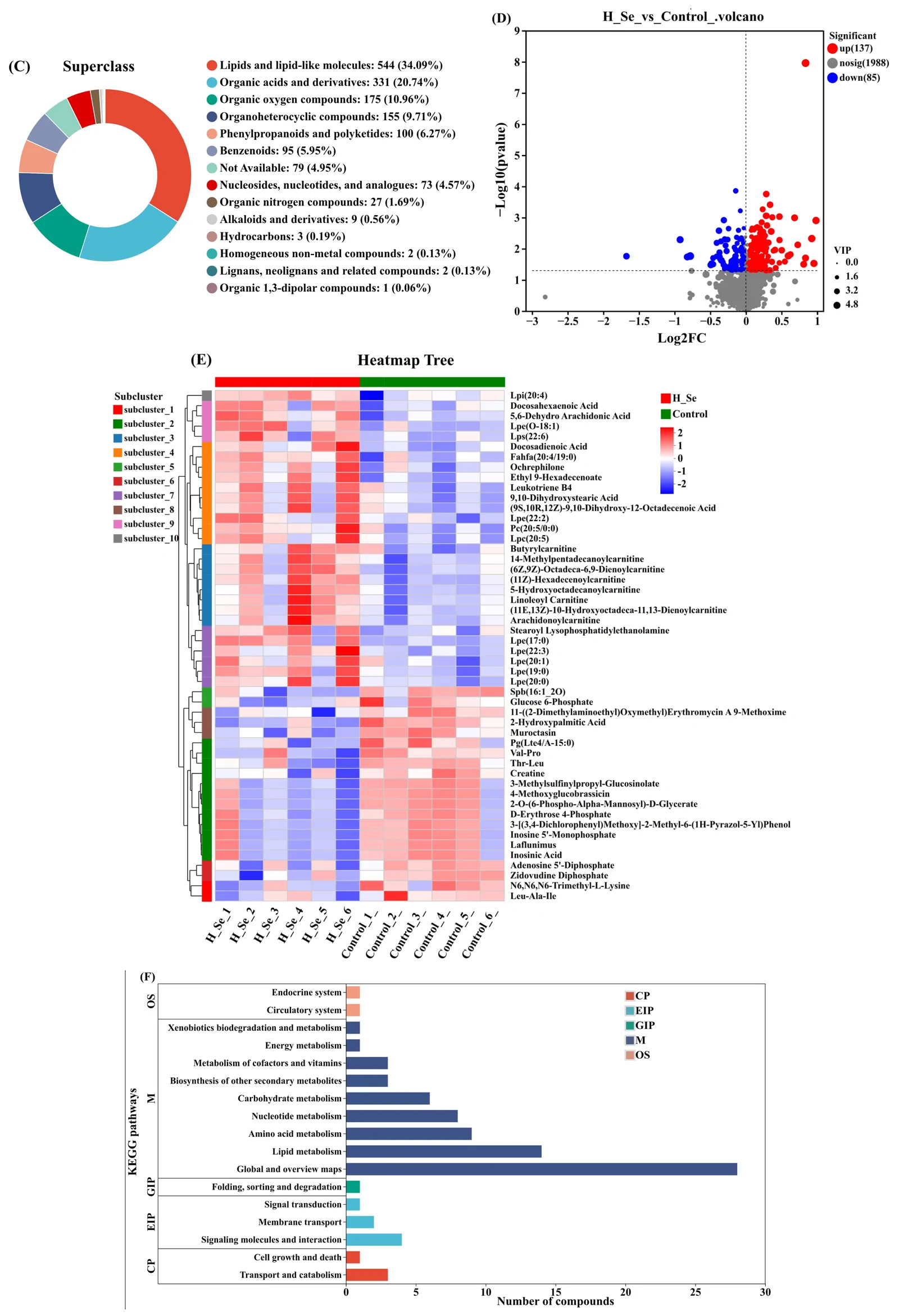

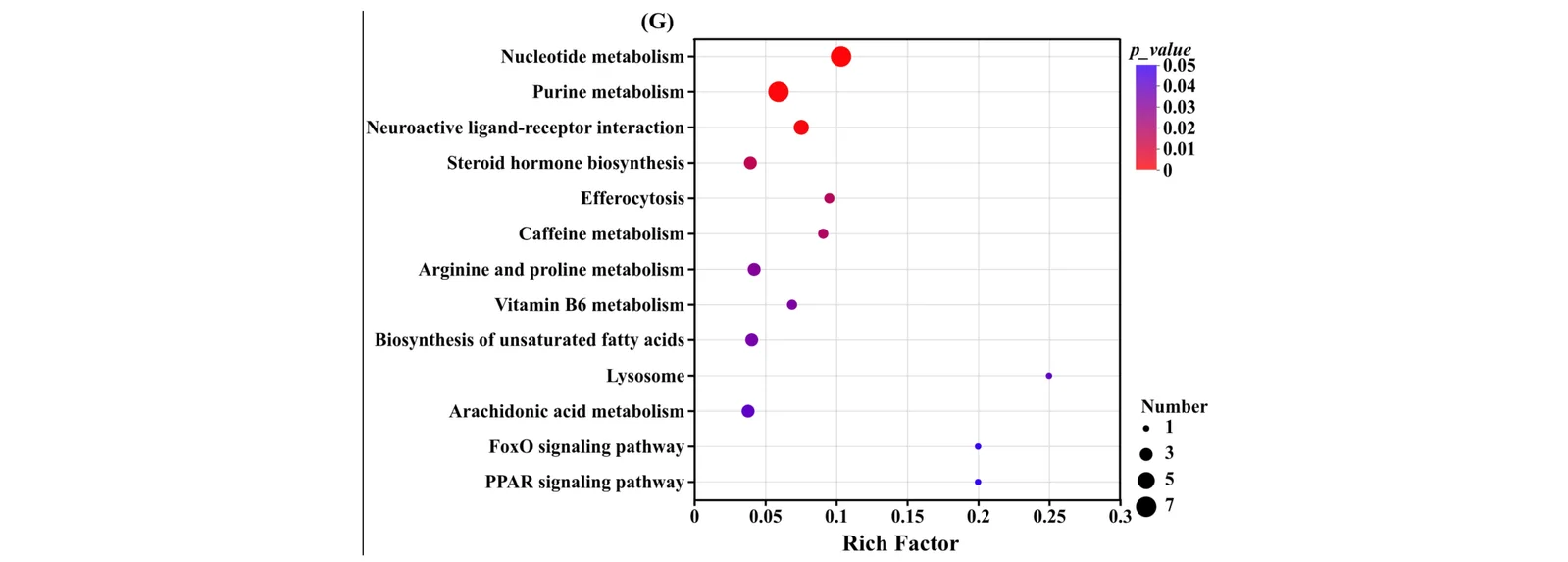
The untargeted metabolomic analysis of common carp dorsal muscle between the Control_ (0 mg/kg Se-supplemented group) and H_Se (2.0 mg/kg Se-supplemented group). (**A**) PLS-DA score plot and (**B**) Venn diagram of metabolites. (**C**) HMDB compound classification of metabolites. (**D**) Volcano plot of differential metabolites. (**E**) Heatmap of differential metabolites. (**F**) Classification map of the KEGG pathway related to the differential metabolites. (**G**) Bubble plot of top enriched KEGG pathways.

**Table 1:** Muscle fatty acid composition of adult common carp (*Cyprinus carpio*) fed diets supplemented with graded levels of sodium selenite. Data are expressed as means ± SD (*n* = 3 cages per group. For each cage, tissues from 4 individual fish were pooled to form one composite sample for analytical measurement. Values in the same row with different superscript letters differ significantly (*p* < 0.05).

Fatty Acids	Selenium Concentration (mg/kg)			
(%)	0.0	0.5	1.0	2.0
C14:0	0.37 ± 0.01 ^a^	0.31 ± 0.06 ^a^	0.32 ± 0.05 ^a^	0.30 ± 0.02 ^a^
C15:0	0.12 ± 0.00 ^a^	0.13 ± 0.01 ^a^	0.13 ± 0.01 ^a^	0.13 ± 0.01 ^a^
C16:0	14.76 ± 0.02 ^b^	14.38 ± 0.18 ^a^	14.90 ± 0.21 ^b^	15.32 ± 0.31 ^c^
C16:1	1.50 ± 0.10 ^a^	1.63 ± 0.47 ^a^	1.67 ± 0.37 ^a^	1.98 ± 0.53 ^a^
C17:0	0.15 ± 0.00 ^a^	0.15 ± 0.01 ^ab^	0.17 ± 0.02 ^ab^	0.18 ± 0.01 ^c^
C17:1	0.11 ± 0.00 ^a^	0.11 ± 0.00 ^ab^	0.12 ± 0.01 ^b^	0.12 ± 0.00 ^b^
C18:0	4.48 ± 0.23 ^a^	4.35 ± 0.36 ^a^	4.52 ± 0.24 ^a^	4.49 ± 0.07 ^a^
C18:1	25.64 ± 0.18 ^a^	26.17 ± 1.77 ^a^	25.29 ± 1.99 ^a^	25.22 ± 0.96 ^a^
C18:2	34.92 ± 0.23 ^b^	35.06 ± 0.62 ^b^	33.94 ± 0.68 ^ab^	33.65 ± 0.66 ^a^
C18:3n6	0.87 ± 0.07 ^a^	0.81 ± 0.12 ^a^	0.80 ± 0.03 ^a^	0.80 ± 0.01 ^a^
C18:3n3	2.21 ± 0.06 ^a^	2.25 ± 0.08 ^a^	2.17 ± 0.04 ^a^	2.16 ± 0.12 ^a^
C20:0	0.23 ± 0.02 ^a^	0.21 ± 0.02 ^a^	0.23 ± 0.02 ^a^	0.23 ± 0.02 ^a^
C20:1	1.67 ± 0.10 ^a^	1.37 ± 0.24 ^a^	1.44 ± 0.20 ^a^	1.45 ± 0.06 ^a^
C20:2	1.25 ± 0.04 ^b^	1.14 ± 0.07 ^a^	1.16 ± 0.05 ^ab^	1.12 ± 0.04 ^a^
C20:3n6	2.73 ± 0.08 ^b^	2.41 ± 0.24 ^a^	2.42 ± 0.13 ^a^	2.47 ± 0.05 ^ab^
C20:3n3	0.10 ± 0.01 ^a^	0.09 ± 0.01 ^a^	0.09 ± 0.01 ^a^	0.10 ± 0.00 ^a^
C20:4	5.16 ± 0.50 ^a^	5.74 ± 0.94 ^a^	5.84 ± 0.70 ^a^	5.05 ± 0.73 ^a^
C20:5	0.42 ± 0.06 ^a^	0.41 ± 0.05 ^a^	0.58 ± 0.10 ^b^	0.61 ± 0.08 ^b^
C22:0	0.08 ± 0.03 ^a^	0.08 ± 0.01 ^a^	0.08 ± 0.01 ^a^	0.07 ± 0.00 ^a^
C22:1	0.09 ± 0.01 ^a^	0.08 ± 0.00 ^a^	0.07 ± 0.00 ^a^	0.08 ± 0.00 ^a^
C22:6	3.13 ± 0.16 ^a^	3.12 ± 0.48 ^a^	3.84 ± 0.87 ^ab^	4.48 ± 0.54 ^c^
∑SFA	20.20 ± 0.27 ^b^	19.54 ± 0.50 ^a^	20.37 ± 0.15 ^b^	20.81 ± 0.23 ^b^
∑UFA	79.80 ± 0.27 ^a^	80.46 ± 0.50 ^b^	79.63 ± 0.15 ^a^	79.19 ± 0.23 ^a^
∑MUFA	29.01 ± 0.38 ^a^	29.75 ± 2.28 ^a^	27.89 ± 1.22 ^a^	28.35 ± 0.76 ^a^
∑PUFA	50.79 ± 0.11 ^a^	50.71 ± 1.78 ^a^	51.74 ± 1.10 ^a^	50.84 ± 0.54 ^a^
EPA + DHA	3.55 ± 0.09 ^a^	3.46 ± 0.48 ^a^	4.78 ± 0.39 ^b^	5.14 ± 0.61 ^b^
AI	1.86 ± 0.04 ^a^	1.53 ± 0.31 ^a^	1.47 ± 0.16 ^a^	1.47 ± 0.55 ^a^

Note: SFA, saturated fatty acid. UFA, unsaturated fatty acid. MUFA, monounsaturated fatty acid. PUFA, polyunsaturated fatty acids. EPA, eicosapentaenoic acid, C20:5. DHA, docosahexaenoic acid, C22:6. AI, atherosclerosis index.

**Table 2:** Free amino acid profiles and taste-active components in muscle of adult common carp (*Cyprinus carpio*) fed diets supplemented with graded levels of sodium selenite. Data are expressed as means ± SD (*n* = 3 cages per group). For each cage, tissues from 4 individual fish were pooled to form one composite sample for analytical measurement. Values in the same row with different superscript letters differ significantly (*p* < 0.05).

Amino Acids (mg/100 g)	Se Concentration (mg/kg)				Taste Attribute
	0.0	0.5	1.0	2.0	
Asp	4.46 ± 2.09 ^a^	4.28 ± 2.53 ^a^	5.44 ± 1.63 ^a^	4.19 ± 0.66 ^a^	Umami (+)
Glu	13.16 ± 2.19 ^a^	17.95 ± 1.73 ^ab^	21.65 ± 3.64 ^b^	21.69 ± 2.46 ^b^	Umami (+)
Gly	110.48 ± 8.35 ^a^	119.90 ± 3.18 ^ab^	117.23 ± 4.96 ^a^	131.22 ± 8.64 ^b^	Sweet (+)
Ala	24.69 ± 6.08 ^a^	23.40 ± 1.41 ^a^	20.04 ± 2.77 ^a^	23.76 ± 1.05 ^a^	Sweet (+)
Thr	13.08 ± 1.33 ^a^	9.18 ± 1.18 ^b^	9.41 ± 0.52 ^b^	11.42 ± 1.68 ^ab^	Sweet (+)
Ser	4.93 ± 1.59 ^a^	4.53 ± 2.79 ^a^	5.03 ± 1.63 ^a^	5.47 ± 2.21 ^a^	Sweet (+)
Pro	36.72 ± 7.27 ^ab^	24.24 ± 2.13 ^a^	38.68 ± 4.04 ^b^	35.34 ± 10.32 ^ab^	Sweet/Bitter (+)
Arg	9.63 ± 4.25 ^a^	6.61 ± 0.91 ^a^	7.94 ± 4.36 ^a^	6.50 ± 2.93 ^a^	Bitter/Sweet (+)
His	292.40 ± 13.24 ^a^	297.72 ± 38.52 ^a^	287.41 ± 14.86 ^a^	293.38 ± 19.61 ^a^	Bitter (−)
Phe	3.89 ± 0.49 ^a^	3.50 ± 0.37 ^a^	3.36 ± 0.37 ^a^	3.62 ± 0.42 ^a^	Bitter (−)
Tyr	3.67 ± 0.79 ^a^	3.76 ± 1.25 ^a^	3.01 ± 0.23 ^a^	2.38 ± 1.65 ^a^	Bitter (−)
Val	4.01 ± 0.59 ^a^	3.83 ± 0.69 ^a^	3.59 ± 0.44 ^a^	4.39 ± 0.43 ^a^	Sweet/bitter (−)
Met	1.53 ± 0.54 ^ab^	1.22 ± 0.17 ^a^	1.51 ± 0.08 ^ab^	2.35 ± 0.74 ^b^	Bitter/sweet (−)
Lys	25.25 ± 3.66 ^a^	21.18 ± 0.98 ^a^	26.83 ± 10.49 ^a^	20.63 ± 2.75 ^a^	Sweet/bitter (−)
Ile	3.45 ± 0.58 ^a^	3.23 ± 1.12 ^a^	3.41 ± 0.49 ^a^	3.86 ± 0.38 ^a^	Bitter (−)
Leu	7.50 ± 1.23 ^a^	6.72 ± 0.88 ^a^	6.87 ± 0.68 ^a^	6.91 ± 1.05 ^a^	Bitter (−)
Trp	5.71 ± 0.83 ^a^	4.94 ± 2.92 ^a^	5.50 ± 1.25 ^a^	6.98 ± 0.75 ^a^	Bitter (−)
UAAs	17.62 ± 4.28 ^a^	22.23 ± 4.12 ^ab^	27.09 ± 2.44 ^b^	25.89 ± 1.88 ^b^	Umami (+)
SAAs	199.53 ± 4.75 ^ab^	187.85 ± 6.53 ^a^	198.33 ± 11.18 ^ab^	213.70 ± 8.25 ^b^	Sweet (+)
BAAs	347.41 ± 14.72 ^a^	346.10 ± 39.18 ^a^	341.50 ± 6.15 ^a^	344.51 ± 20.41 ^a^	Bitter (−)
TFAAs	564.56 ± 21.75 ^a^	556.19 ± 37.37 ^a^	566.92 ± 7.67 ^a^	584.10 ± 29.51 ^a^	−

Note: UAAs, umami amino acids, the total of Asp and Glu; SAAs, sweet amino acids, the total of Thr, Ser, Gly, Ala, Arg and Pro; BAAs, bitter amino acids, the total of Val, Met, Ile, Leu, Phe, Tyr, Lys, His, and Trp. TFAAs, total free amino acids; (+), pleasant; (−), unpleasant.

## Data Availability

The original contributions presented in this study are included in the article/Supplementary Material. Further inquiries can be directed to the corresponding author.

## Supplementary Materials

The following supporting information can be downloaded at: https://www.mdpi.com/article/10.3390/antiox15101233/s1, Figure S1: Second-order polynomial regression analysis of optimum dietary selenium requirement of adult common carp (*Cyprinus carpio*) based on weight gain rate (A) and specific growth rate (B). Data are expressed as means ± SD (*n* = 3 cages per group).; Figure S2: Effect of dietary sodium selenite levels on the proximate composition of adult common carp (*Cyprinus carpio*) muscle: moisture (A), ash (B), crude fat (C), and crude protein (D). Data are expressed as means ± SD (*n* = 3 cages per group). For each cage, tissues from 4 individual fish were pooled to form one composite sample for analytical measurement. Bars with different letters are significantly different (*p* < 0.05).; Figure S3: Permutation test of the PLS-DA model.; Table S1: Basic formula of the experimental feed.; Table S2: Growth performance of common carp (*Cyprinus carpio*) fed with different sodium selenite concentrations for 8 weeks. Data are expressed as means ± SD (*n* = 3 cages per group). Values in the same row with different superscript letters differ significantly (*p* < 0.05).; Table S3: Effect of dietary sodium selenite on the hydrolyzed amino acid compositions of common carp (*Cyprinus carpio*) muscle. Data are expressed as means ± SD (*n* = 3 cages per group). For each cage, tissues from 4 individual fish were pooled to form one composite sample for analytical measurement. Values in the same row with different superscript letters differ significantly (*p* < 0.05).; Table S4: Identification of volatile compounds in the muscle of common carp (*Cyprinus carpio*) fed with different sodium selenite. Data are expressed as means ± SD (*n* = 3 cages per group). For each cage, tissues from 4 individual fish were pooled to form one composite sample for analytical measurement. Values in the same row with different superscript letters differ significantly (*p* < 0.05).; Table S5: Summary of one-way ANOVA results for all measured variables.
