# Supplementary material for: Effects of Short-Term Dietary Sodium Selenite Supplementation on Antioxidant Status, Heavy Metal Deposition, Flesh Quality and Flavor-Related Compounds of Common Carp (Cyprinus carpio), and a Potential Mechanism
Source: Antioxidants (Basel). 2026 Sep 24;15(10):1233. doi: 10.3390/antiox15101233 (PMC13700859; doi:10.3390/antiox15101233)
Supplement: Supplementary file 1 [file antioxidants-15-01233-s001.zip › antioxidants-4528359-supplementary.pdf]

Supplementary material

Figure S1 Second-order polynomial regression analysis of optimum dietary selenium requirement of adult common carp (*Cyprinus carpio*) based on weight gain rate (A) and specific growth rate (B). Data are expressed as means ± SD (n = 3 cages per group).

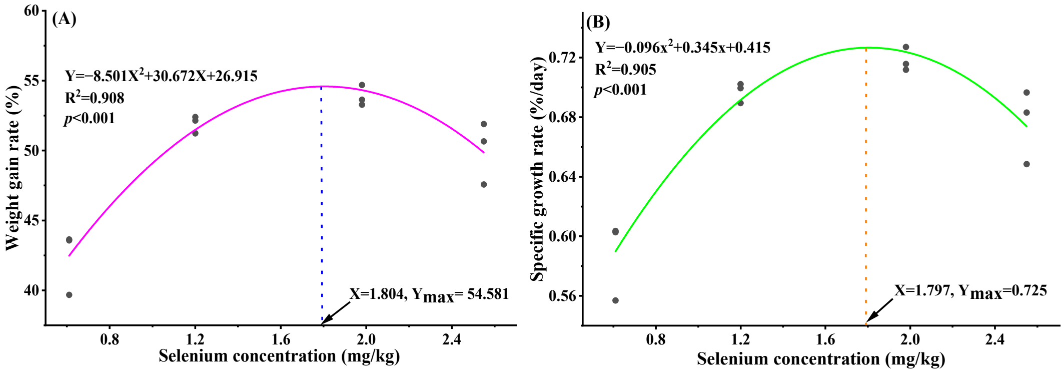

Figure S2 Effects of dietary sodium selenite levels on the proximate composition of adult common carp (*Cyprinus carpio*) muscle: moisture (A), ash (B), crude fat (C), and crude protein (D). Data are expressed as means ± SD (n = 3 cages per group). For each cage, tissues from 4 individual fish were pooled to form one composite sample for analytical measurement. Bars with different letters are significantly different ($p < 0.05$).

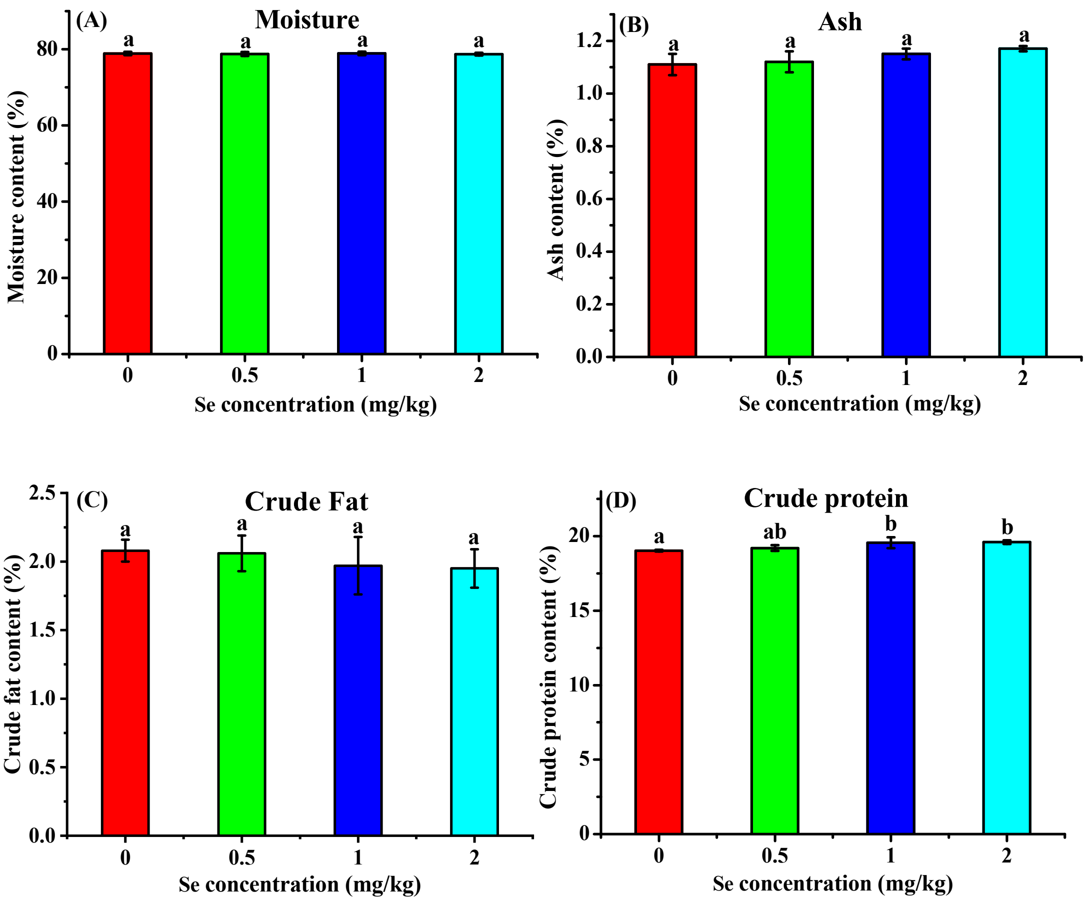

Figure S3 Permutation test of the PLS-DA model.

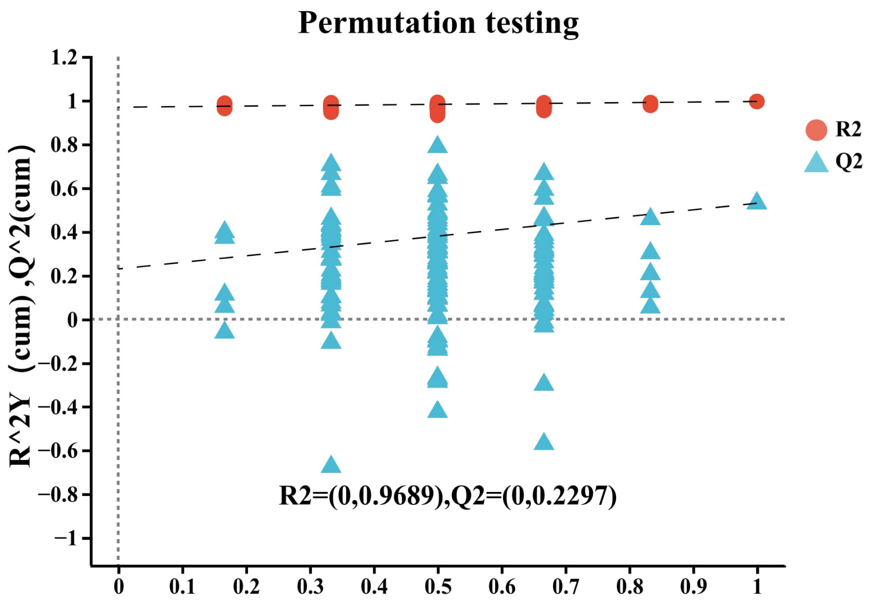

Table S1 Basic formula of the experimental feed

Ingredients	Inclusion level (g)
Type 200 rapeseed meal	180
43% soybean meal	315
Peanut meal	45
Palm meal	90
Flour grade 1	60
Bran level 1	75
Spout corn husks	70
Domestic DDGS (1stgrade)	30
Rice bran	40
Soybean oil grade 3	5
Bentonite secondary	45
Calcium dihydrogen phosphate	18
Propionic acid type solid protection agent	0.3
Sodium chloride (96.5%)	2
L-ascorbate-2-phosphate	0.3
Polysaccharide complex premixed	1
Flavoring agent for aquatic products	1
L-catnitine hydrochloride (Liver cholesterin)	0.4
Vitamin premix feed	2
Microelement premix feed for omnivorous fish	20

Table S2 Growth performance of common carp (*Cyprinus carpio*) fed with different sodium selenite concentrations for 8 weeks. Data are expressed as means ± SD (n = 3 cages per group). Values in the same row with different superscript letters differ significantly ($p < 0.05$).

Parameter	Selenium concentration (mg/kg)			
	0.0	0.5	1.0	2.0
WGR (%)	$42.29±2.27^{a}$	$51.92±0.61^{bc}$	$53.87±0.73^{c}$	$50.03±2.23^{b}$
SGR (%/day)	$0.58±0.03^{a}$	$0.70±0.01^{bc}$	$0.72±0.01^{c}$	$0.68±0.02^{b}$
HSI (%)	$2.66±0.40^{a}$	$2.79±0.21^{a}$	$2.91±0.29^{a}$	$2.96±0.46^{a}$
VSI (%)	$20.52±0.05^{a}$	$22.92±3.83^{a}$	$20.97±0.49^{a}$	$17.52±4.21^{a}$
CF (g/cm^3)	$1.50±0.07^{a}$	$1.46±0.06^{a}$	$1.53±0.10^{a}$	$1.38±0.20^{a}$
Survival rate (%)	100	100	100	100

Table S3 Effect of dietary sodium selenite on the hydrolyzed amino acid compositions of common carp (*Cyprinus carpio*) muscle. Data are expressed as means ± SD (n = 3 cages per group). For each cage, tissues from 4 individual fish were pooled to form one composite sample for analytical measurement. Values in the same row with different superscript letters differ significantly ($p < 0.05$).

Amino acids	Se concentration (mg/kg)			
(g/100 g)	0.0	0.5	1.0	2.0
Asp	1.77±0.07^{a}	1.79±0.04^{a}	1.84±0.09^{a}	1.84±0.11^{a}
Glu	2.77±0.09^{a}	2.76±0.07^{a}	2.88±0.15^{a}	2.85±0.21^{a}
Ser	0.61±0.02^{a}	0.61±0.03^{a}	0.64±0.03^{a}	0.63±0.04^{a}
His	0.78±0.04^{ab}	0.76±0.08^{a}	0.76±0.03^{b}	0.77±0.06^{ab}
Gly	0.91±0.01^{a}	0.87±0.04^{a}	0.91±0.04^{a}	0.93±0.07^{a}
Thr	0.71±0.03^{a}	0.72±0.03^{a}	0.75±0.04^{a}	0.75±0.05^{a}
Arg	1.00±0.05^{a}	0.99±0.06^{a}	1.04±0.05^{a}	1.03±0.07^{a}
Ala	0.99±0.04^{a}	0.98±0.05^{a}	1.02±0.05^{a}	1.03±0.07^{a}
Tyr	0.49±0.05^{a}	0.49±0.03^{a}	0.49±0.01^{a}	0.52±0.04^{a}
Cys	0.06±0.01^{a}	0.06±0.00^{a}	0.07±0.00^{a}	0.07±0.01^{a}
Val	0.92±0.06^{a}	0.92±0.03^{a}	0.96±0.05^{a}	0.95±0.05^{a}
Met	0.44±0.05^{a}	0.43±0.06^{a}	0.48±0.04^{a}	0.50±0.05^{a}
Phe	0.71±0.04^{a}	0.73±0.02^{a}	0.75±0.04^{a}	0.76±0.05^{a}
Ile	0.83±0.04^{a}	0.85±0.04^{a}	0.88±0.05^{a}	0.87±0.06^{a}
Leu	1.34±0.06^{a}	1.37±0.06^{a}	1.42±0.07^{a}	1.42±0.09^{a}
Lys	1.58±0.09^{a}	1.60±0.07^{a}	1.68±0.08^{a}	1.66±0.11^{a}
Pro	0.70±0.27^{a}	0.72±0.13^{a}	0.84±0.09^{a}	0.77±0.09^{a}
TAA	16.57±0.65^{a}	16.57±0.72^{a}	17.32±0.85^{a}	17.28±1.11^{a}
EAA	6.55±0.34^{a}	6.60±0.32^{a}	6.92±0.35^{a}	6.90±0.44^{a}
NEAA	10.02±0.04^{a}	9.96±0.41^{a}	10.41±0.50^{a}	10.38±0.67^{a}
EAA/NEAA	0.65±0.02^{a}	0.66±0.01^{a}	0.66±0.01^{a}	0.66±0.00^{a}

Note: TAA, total amino acids. EAA, essential amino acids, the total of Thr, Val, Met, Phe, Ile, Leu, and Lys. NEAA, non-essential amino acids, the total of Asp, Glu, Ser, His, Gly, Cys, and Pro.

Table S4 Identification of volatile compounds in the muscle of common carp (*Cyprinus carpio*) fed with different sodium selenite levels. Data are expressed as means ± SD (n = 3 cages per group). For each cage, tissues from 4 individual fish were pooled to form one composite sample for analytical measurement. Values in the same row with different superscript letters differ significantly ($p < 0.05$).

No.	Compounds	Relative content (2,4,6-trimethylpyridine equivalents, μg/kg)			
		Control	0.5 Se mg/kg	1.0 Se mg/kg	2.0 Se mg/kg
	**Aldehydes (8)**				
1	2-Octenal, (E)-	37.09±5.49^{a}	160.04±21.84^{b}	277.24±26.45^{c}	153.82±20.51^{b}
2	Hexanal	2958.16±279.08^{a}	2580.51±263.95^{a}	6231.02±842.97^{b}	8362.66±119.62^{c}
3	Nonanal	160.38±27.41^{a}	438.98±54.66^{b}	647.09±94.67^{c}	411.92±42.88^{b}
4	Benzaldehyde	133.95±11.23^{c}	77.07±10.29^{a}	124.39±10.79^{c}	100.31±16.03^{b}
5	Hexadecanal	6.30±0.95^{a}	16.96±1.13^{c}	13.18±2.05^{b}	12.02±1.31^{b}
6	Octanal	60.53±9.56^{a}	141.67±10.52^{b}	186.90±11.38^{c}	129.54±10.37^{b}
7	2,4-Nonadienal, (E,E)-	8.15±1.00^{a}	31.34±4.76^{b}	52.32±7.11^{c}	33.47±5.07^{b}
8	Tetradecanal	3.86±0.53^{a}	7.42±1.00^{b}	13.55±1.81^{c}	7.38±1.10^{b}
	Total	3368.43±285.20^{a}	3453.98±318.64^{a}	7612.36±75.33^{b}	9211.13±82.30^{c}
	**Alcohol (8)**				
9	1-Hexanol	125.70±20.03^{a}	142.90±20.65^{ab}	269.60±33.03^{c}	179.30±16.28^{b}
10	1-Pentanol	84.01±17.24^{a}	205.85±36.50^{b}	376.31±55.56^{c}	259.14±30.32^{b}
11	1-Hexanol, 2-ethyl-	12.00±1.47^{ab}	9.25±2.33^{a}	14.60±1.81^{b}	10.32±1.32^{a}
12	Phenylethyl Alcohol	33.69±0.75^{d}	25.32±1.60^{c}	21.07±3.36^{b}	14.74±0.54^{a}

13	2-Octen-1-ol, (E)-	5.59±0.91[a]	23.30±3.26[b]	60.01±3.18[d]	33.57±4.72[c]
14	1-Penten-3-ol	46.00±3.41[a]	66.85±16.99[a]	136.41±15.16[b]	130.13±14.51[b]
15	2-Penten-1-ol, (Z)-	4.16±0.61[a]	14.59±2.40[b]	24.71±3.26[c]	17.26±1.57[b]
16	1-Octanol	20.85±3.38[a]	49.68±3.96[b]	89.16±12.18[c]	49.03±8.28[b]
	Total	332.00±5.64[a]	537.74±66.55[b]	991.88±106.50[d]	693.48±6.72[c]
	**Esters (6)**				
17	Hexadecanoic acid, methyl ester	8.03±1.53[a]	33.55±3.54[c]	28.05±3.29[b]	35.26±1.39[c]
18	Dibutyl phthalate	2.32±0.06[a]	2.67±0.44[ab]	3.47±0.80[b]	2.26±0.36[a]
19	1,2-Benzenedicarboxylic acid, bis(2-methylpropyl) ester	1.84±0.11[a]	1.78±0.34[a]	1.83±0.30[a]	1.48±0.10[a]
20	Methyl tetradecanoate	4.11±0.57[a]	12.53±0.79[b]	13.54±1.13[b]	14.60±1.06[b]
21	Diethyl Phthalate	0.73±0.11[ab]	0.68±0.09[a]	0.94±0.15[b]	0.78±0.13[ab]
22	Carbamodithioic acid, diethyl-, methyl ester	386.98±54.12[b]	441.86±45.84[b]	274.78±23.50[a]	275.54±40.49[a]
	Total	17.03±0.29[a]	51.20±3.34[bc]	47.83±4.41[b]	54.39±1.87[c]
	**Ketone (4)**				
23	3,5-Octadien-2-one, (E,E)-	17.50±2.70[a]	73.26±14.19[b]	153.16±19.34[c]	106.91±14.59[d]
24	Acetophenone	3.06±0.15[a]	3.66±0.65[ab]	4.13±0.66[c]	3.95±0.21[ab]
25	1-Octen-3-one	20.08±3.61[a]	50.37±6.95[b]	84.98±8.56[c]	53.46±9.99[b]
26	3-Octanone	6.55±102[a]	26.69±4.52[b]	48.67±3.88[c]	28.27±2.15[b]
	Total	47.20±5.68[a]	153.98±16.92[b]	290.94±20.58[d]	192.59±13.65[c]
	**Acids (3)**				
27	Hexanoic acid	10.11±1.19[a]	27.82±3.63[b]	46.37±7.28[c]	27.68±4.14[b]

28	Nonanoic acid	7.32±1.20a	10.53±1.10b	12.32±1.04b	7.64±0.86a
29	Octanoic acid	3.17±0.70a	9.14±1.88b	11.40±2.03b	8.95±1.45b
	Total	20.60±3.00a	47.49±3.71b	70.09±6.10c	44.27±6.20b
	**Aromatic (8)**				
30	Benzene, 1,2,3-trimethyl-	28.39±3.46a	98.56±16.222b	121.55±15.58b	111.08±14.01b
31	Biphenyl	0.14±0.03b	0.18±0.04b	0.18±0.05b	0.00±0.00a
32	Phenol	3.04±0.48a	6.29±0.57b	4.43±1.31a	3.14±0.18a
33	Benzene, 1,1'-[oxybis(methylene)]bis-	0.98±0.08a	1.84±0.19b	2.01±0.12b	1.86±0.18b
34	5-Thiazoleethanol, 4-methyl-	4.68±0.06a	9.26±1.08c	9.17±0.44c	7.41±1.37b
35	Furan, 2-pentyl-	20.34±1.67a	23.15±3.92a	93.42±13.05b	77.39±10.34b
36	Benzothiazole	13.52±1.06a	15.05±1.41a	13.02±1.24a	15.55±1.88a
37	Phenol, 2-methoxy-3-(2-propenyl)-	1110.38±162.01b	2554.91±364.86c	370.08±20.56a	313.59±42.45a
	Total	1181.48±164.29b	2709.25±360.31c	613.86±44.15a	530.03±61.52a
	**Alkanes (2)**				
38	Heptadecane	507.08±32.77c	201.64±14.48b	158.02±32.98b	110.98±4.28a
39	Hexadecane	16.32±0.79ab	13.42±2.37a	18.64±3.03b	13.91±2.03a
	Total	261.70±16.76c	107.53±6.88b	88.33±15.92b	62.44±2.89a
	**Amine (1)**				
40	Formamide, N,N-dibutyl-	3.49±0.59a	4.62±0.55ab	7.42±0.98c	5.84±0.79bc
	Total	5231.93±125.49a	7065.79±346.34b	9722.70±838.48c	10794.18±1018.85c

Table S5 Summary of one-way ANOVA results for all measured variables.

Measured variables	F-value	df	p-value
**Growth performance**			
WGR	28.048	3/8	0.000
SGR	27.479	3/8	0.000
HSI	0.432	3/8	0.736
VSI	1.838	3/8	0.218
CF	0.780	3/8	0.538
**Proximate Composition**			
Ash	2.314	3/8	0.153
Moisture	0.112	3/8	0.951
Crude protein	5.026	3/8	0.030
Crude lipid	0.596	3/8	0.636
**Antioxidant enzyme activity in muscle**			
GSH-Px	101.602	3/8	0.000
SOD	11.966	3/8	0.003
CAT	42.727	3/8	0.000
MDA	50.564	3/8	0.000
**Antioxidant enzyme activity in liver**			
GSH-Px	145.881	3/8	0.000
SOD	2.087	3/8	0.180
CAT	6.118	3/8	0.018
MDA	14.496	3/8	0.001
**Se content in muscle**			
Se	11.486	3/8	0.003
**Se content in liver**			
Se	55.020	3/8	0.000
**Metal content in muscle**			
Hg	9.060	3/8	0.006
Cd	20.588	3/8	0.000
**Texture profile analysis**			
Hardness	6.596	3/8	0.015
Springiness	195.755	3/8	0.000
Gumminess	8.664	3/8	0.007

Chewiness	26.158	3/8	0.000
Resilience	8.057	3/8	0.008
**Water holding capacity**			
Centrifugal loss	39.861	3/8	0.000
**Hydrolyzed Amino Acid Composition**			
Asp	0.602	3/8	0.632
Glu	0.504	3/8	0.690
Ser	0.821	3/8	0.518
His	0.080	3/8	0.969
Gly	1.038	3/8	0.426
Thr	0.796	3/8	0.530
Arg	0.723	3/8	0.566
Ala	0.663	3/8	0.597
Tyr	0.443	3/8	0.729
Val	0.496	3/8	0.695
Met	1.200	3/8	0.370
Phe	0.813	3/8	0.522
Ile	0.828	3/8	0.514
Leu	0.848	3/8	0.505
Lys	0.894	3/8	0.485
Pro	0.407	3/8	0.752
TAA	0.752	3/8	0.551
EAA	0.863	3/8	0.498
NEAA	0.648	3/8	0.606
EAA/NEAA	0.534	3/8	0.672
**Fatty Acid Composition**			
C14:0	1.655	3/8	0.253
C15:0	0.393	3/8	0.761
C16:0	10.706	3/8	0.004
C16:1	0.764	3/8	0.545
C17:0	2.806	3/8	0.108
C17:1	4.582	3/8	0.038
C18:0	0.282	3/8	0.837
C18:1	0.236	3/8	0.869
C18:2	4.478	3/8	0.040

C18:3n6	0.595	3/8	0.636
C18:3n3	0.822	3/8	0.517
C20:0	1.103	3/8	0.403
C20:1	1.888	3/8	0.210
C20:2	3.544	3/8	0.068
C20:3n6	3.250	3/8	0.081
C20:3n3	0.899	3/8	0.483
C20:4	0.350	3/8	0.791
C20:5	5.555	3/8	0.023
C22:0	0.049	3/8	0.984
C22:1	1.966	3/8	0.198
C22:6	3.916	3/8	0.054
∑SFA	8.440	3/8	0.007
∑UFA	8.424	3/8	0.007
∑MUFA	1.066	3/8	0.416
∑PUFA	0.597	3/8	0.634
EPA+DHA	11.496	3/8	0.003
AI	2.288	3/8	0.155
**Free Amino Acid Composition**			
Asp	0.284	3/8	0.836
Glu	7.225	3/8	0.012
Gly	5.002	3/8	0.031
Ala	1.036	3/8	0.427
Thr	6.460	3/8	0.016
Ser	0.085	3/8	0.966
Pro	2.798	3/8	0.109
Arg	0.553	3/8	0.660
His	0.095	3/8	0.961
Phe	0.916	3/8	0.476
Tyr	1.002	3/8	0.440
Cys	1.289	3/8	0.343
Val	1.119	3/8	0.397
Met	3.224	3/8	0.082
Lys	0.843	3/8	0.508
Ile	0.432	3/8	0.736

Leu	0.369	3/8	0.778
Trp	0.789	3/8	0.533
UAAs	4.850	3/8	0.033
SAAs	5.239	3/8	0.027
BAAs	0.025	3/8	0.994
TAAs	0.571	3/8	0.650
**Volatile compounds**			
2-Octenal, (E)-	70.915	3/8	0.000
Hexanal	48.487	3/8	0.000
Nonanal	32.814	3/8	0.000
Benzaldehyde	12.912	3/8	0.002
Hexadecanal	28.861	3/8	0.000
Octanal	74.727	3/8	0.000
2,4-Nonadienal, (E,E)-	39.375	3/8	0.000
Tetradecanal	33.637	3/8	0.000
Total aldehydes	54.612	3/8	0.000
1-Hexanol	22.625	3/8	0.000
1-Pentanol	31.335	3/8	0.000
1-Hexanol, 2-ethyl-	5.171	3/8	0.028
Phenylethyl Alcohol	51.649	3/8	0.000
2-Octen-1-ol, (E)-	141.655	3/8	0.000
1-Penten-3-ol	34.527	3/8	0.000
2-Penten-1-ol, (Z)-	45.205	3/8	0.000
1-Octanol	38.759	3/8	0.000
Total alcohol	52.197	3/8	0.000
Hexadecanoic acid, methyl ester	67.910	3/8	0.000
Dibutyl phthalate	3.814	3/8	0.058
1,2-Benzenedicarboxylic acid, bis(2-methylpropyl) ester	1.525	3/8	0.281
Methyl tetradecanoate	82.491	3/8	0.000
Diethyl Phthalate	2.690	3/8	0.117
Carbamodithioic acid, diethyl-, methyl ester	11.575	3/8	0.003
Total ester	92.757	3/8	0.000
3,5-Octadien-2-one, (E,E)-	49.201	3/8	0.107
Acetophenone	2.820	3/8	0.000

1-Octen-3-one	36.041	3/8	0.000
3-Octanone	86.306	3/8	0.000
Total ketone	131.255	3/8	0.000
Hexanoic acid	31.088	3/8	0.000
Nonanoic acid	15.411	3/8	0.001
Octanoic acid	14.424	3/8	0.001
Total acid	50.044	3/8	0.000
Benzene, 1,2,3-trimethyl-	30.131	3/8	0.000
Biphenyl	15.741	3/8	0.001
Phenol	11.959	3/8	0.003
Benzene, 1,1'-[oxybis(methylene)]bis-	30.569	3/8	0.000
5-Thiazoleethanol, 4-methyl-	16.978	3/8	0.001
Furan, 2-pentyl-	56.621	3/8	0.000
Benzothiazole	2.134	3/8	0.174
Phenol, 2-methoxy-3-(2-propenyl)-	80.882	3/8	0.000
Total aromatic compound	75.222	3/8	0.000
Heptadecane	160.883	3/8	0.000
Hexadecane	3.552	3/8	0.067
Total alkane	163.803	3/8	0.000
Formamide, N, N-dibutyl-	15.287	3/8	0.001
Total volatile compound	40.806	3/8	0.000
Aldehyde percentage	14.495	3/8	0.001
Aromatic compound percentage	81.631	3/8	0.000
Alcohol percentage	15.023	3/8	0.001
Alkane percentage	356.900	3/8	0.000
Ketone percentage	69.688	3/8	0.000
Acid percentage	26.557	3/8	0.000
Ester percentage	51.773	3/8	0.000
Amine percentage	2.885	3/8	0.103
